# Structures and therapeutic potential of anti-RBD human monoclonal antibodies against SARS-CoV-2

**DOI:** 10.7150/thno.65563

**Published:** 2022-01-01

**Authors:** Kuan-Ying A. Huang, Daming Zhou, Tiong Kit Tan, Charles Chen, Helen M. E. Duyvesteyn, Yuguang Zhao, Helen M. Ginn, Ling Qin, Pramila Rijal, Lisa Schimanski, Robert Donat, Adam Harding, Javier Gilbert-Jaramillo, William James, Julia A. Tree, Karen Buttigieg, Miles Carroll, Sue Charlton, Chia-En Lien, Meei-Yun Lin, Cheng-Pin Chen, Shu-Hsing Cheng, Xiaorui Chen, Tzou-Yien Lin, Elizabeth E. Fry, Jingshan Ren, Che Ma, Alain R. Townsend, David I. Stuart

**Affiliations:** 1 Division of Pediatric Infectious Diseases, Department of Pediatrics, Chang Gung Memorial Hospital, Taoyuan, Taiwan; 2 Research Center for Emerging Viral Infections, College of Medicine, Chang Gung University, Taoyuan, Taiwan; 3 Genomics Research Center, Academia Sinica, Taipei, Taiwan; 4 Division of Structural Biology, Nuffield Department of Medicine, University of Oxford, The Wellcome Centre for Human Genetics, Headington, Oxford, UK; 5 MRC Human Immunology Unit, Weatherall Institute of Molecular Medicine, University of Oxford, John Radcliffe Hospital, Oxford, UK; 6 Medigen Vaccine Biologics Corporation, Taipei, Taiwan; 7 Temple University, Philadelphia, PA 19122, USA; 8 Diamond Light Source Ltd, Harwell Science & Innovation Campus, Didcot, UK; 9 Sir William Dunn School of Pathology, South Park Road, University of Oxford, UK; 10 National Infection Service, Public Health England, Porton Down, Salisbury, UK; 11 Institute of Public Health, National Yang-Ming Chiao Tung University, Taipei, Taiwan; 12 Department of Infectious Diseases, Taoyuan General Hospital, Ministry of Health and Welfare, Taoyuan, and National Yang-Ming University, Taipei, Taiwan; 13 Department of Infectious Diseases, Taoyuan General Hospital, Ministry of Health and Welfare, Taoyuan, and Taipei Medical University, Taipei, Taiwan

**Keywords:** SARS-CoV-2, Human monoclonal antibody, *In vitro* and *in vivo* function, Antibody-antigen complex, Receptor-binding domain epitope, Antibody cocktail

## Abstract

**Background:** Administration of potent anti-receptor-binding domain (RBD) monoclonal antibodies has been shown to curtail viral shedding and reduce hospitalization in patients with SARS-CoV-2 infection. However, the structure-function analysis of potent human anti-RBD monoclonal antibodies and its links to the formulation of antibody cocktails remains largely elusive.

**Methods:** Previously, we isolated a panel of neutralizing anti-RBD monoclonal antibodies from convalescent patients and showed their neutralization efficacy *in vitro*. Here, we elucidate the mechanism of action of antibodies and dissect antibodies at the epitope level, which leads to a formation of a potent antibody cocktail.

**Results:** We found that representative antibodies which target non-overlapping epitopes are effective against wild type virus and recently emerging variants of concern, whilst being encoded by antibody genes with few somatic mutations. Neutralization is associated with the inhibition of binding of viral RBD to ACE2 and possibly of the subsequent fusion process. Structural analysis of representative antibodies, by cryo-electron microscopy and crystallography, reveals that they have some unique aspects that are of potential value while sharing some features in common with previously reported neutralizing monoclonal antibodies. For instance, one has a common VH 3-53 public variable region yet is unusually resilient to mutation at residue 501 of the RBD. We evaluate the *in vivo* efficacy of an antibody cocktail consisting of two potent non-competing anti-RBD antibodies in a Syrian hamster model. We demonstrate that the cocktail prevents weight loss, reduces lung viral load and attenuates pulmonary inflammation in hamsters in both prophylactic and therapeutic settings. Although neutralization of one of these antibodies is abrogated by the mutations of variant B.1.351, it is also possible to produce a bi-valent cocktail of antibodies both of which are resilient to variants B.1.1.7, B.1.351 and B.1.617.2.

**Conclusions:** These findings support the up-to-date and rational design of an anti-RBD antibody cocktail as a therapeutic candidate against COVID-19.

## Introduction

In late 2019, a novel coronavirus was identified as the causative agent of a pneumonia cluster in China 1. The virus rapidly spread within China, followed by a global pandemic. In February 2020, the World Health Organization designated the virus severe acute respiratory syndrome coronavirus 2 (SARS-CoV-2) and the disease caused by SARS-CoV-2 is designated as COVID-19 2. The ongoing SARS-CoV-2 pandemic has led to over 236 million confirmed cases and over 4.8 million deaths around the world (https://www.who.int). A number of vaccines have been approved but remain unavailable in many countries 3. There are no specific antiviral drugs available at present. Conservative management and steroid therapy are still considered the mainstay of treatment for SARS-CoV-2 infection.

Passive immunotherapy with convalescent plasma or monoclonal antibody preparations has been evaluated for the treatment of COVID-19 4-7. The trimeric spike glycoprotein on the viral surface is the prime antibody target since the spike plays an essential role in allowing the virus to attach to and infect host cells 8,9. The SARS-CoV-2 spike glycoprotein is composed of domains S1 and S2. The S1 domain contains the receptor-binding domain (RBD) that specifically binds to the cell receptor human angiotensin-converting enzyme 2 (ACE2) and some RBD-bound antibodies block the interaction between the RBD and ACE2 receptor, which lead to the neutralization of SARS-CoV-2 infection 10. Evidence has shown that potently neutralizing monoclonal antibodies that recognize the viral RBD are often elicited in SARS-CoV-2 infection 11. In recent years, highly specific and neutralizing monoclonal antibodies have been successfully isolated against several viruses, serving as an advanced replacement for convalescent plasma in the passive immunotherapy 12, 13. These biologic therapies are now being considered for combating COVID-19 outbreaks (www.fda.gov).

Monoclonal antibodies that target SARS-CoV-2 RBD are being evaluated in outpatients 4, 7, and early trial data suggest that an antibody cocktail of two antibodies, REGN10933 and REGN10987, administered together, reduces viral load and infection-related hospital visits in COVID-19 patients when compared to placebo 7. Recently, an emergency use authorization for the antibody combination has been issued by U.S. Food and Drug Administration for non-hospitalized COVID-19 patients who have certain risk factors for severe disease (www.fda.gov).

Previously, we isolated a panel of anti-spike monoclonal antibodies (mAb) that target a diverse spectrum of epitopes on the spike protein, of which a majority of the RBD-targeting antibodies potently neutralize SARS-CoV-2 and recognize non-overlapping epitopes on the RBD 14. Here, we further investigate the mechanisms of neutralization, *in vivo* efficacy of an antibody cocktail against wild type virus, and delineate the functional epitopes based on antigen-antibody complex structures. The *in vitro* and *in vivo* efficacy of this antibody cocktail clearly indicates the potential of potent anti-RBD antibodies impacting the treatment and prevention of SARS-CoV-2 infection. Finally, we investigate the effects of B.1.1.7, B.1.351 and B.1.617.2 variants of concern on the neutralization properties of these antibodies and explain these effects in terms of their structures.

## Results

### Identification of anti-SARS-CoV-2 RBD antibodies with non-overlapping neutralization sites

**Figure 1:**
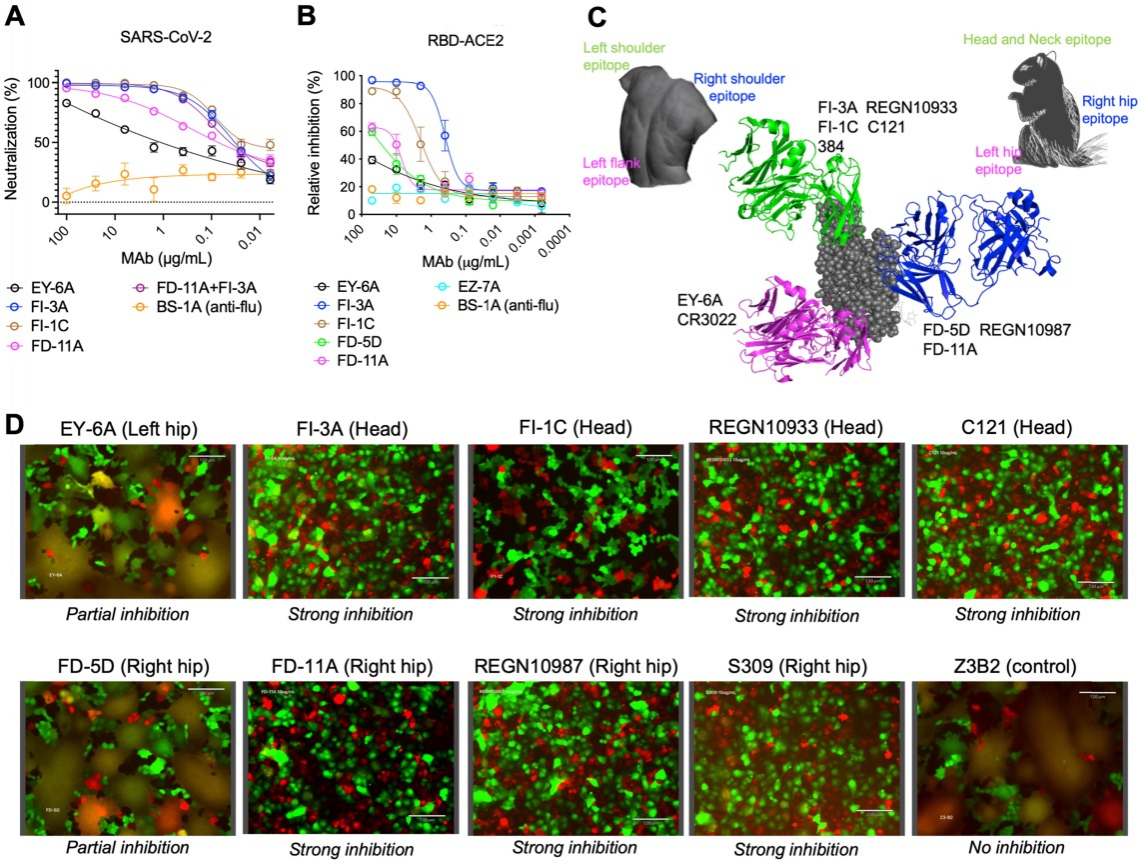
** Functional grouping of neutralizing anti-RBD human monoclonal antibodies. A**, Neutralization of anti-RBD human antibodies against the SARS-CoV-2 (Wuhan) pseudovirus. BS-1A is an anti-influenza H3 human antibody and included as a control. The data represents the mean ± the standard error of mean with three replicates. The 50% inhibitory concentrations of EY-6A, FI-3A, FI-1C, FD-11A and FI-3A plus FD-11A against the virus were 2.110, 0.033, 0.023, 0.060 and 0.035 µg/mL, respectively. **B**, Levels of anti-RBD antibodies that block the binding of biotinylated RBD to ACE2 protein expressed on the MDCK cell, measured in the flow cytometry. The data represents the mean ± the standard error of mean with two replicates.** C**, The epitope grouping of anti-RBD antibodies based on cross-competition analysis and the inhibitory activity of the binding of RBD to the ACE2 protein. The epitopes were designated as the head/neck, left hip and right hip of the “squirrel” RBD 14 equivalent to the left shoulder, left flank and right shoulder epitopes in the “torso” RBD 11. The RBD was shown as spheres in grey (PDB ID, 6XDG) and anti-RBD Fabs REGN10933 (PDB ID, 6XDG), REGN10987 (PDB ID, 6XDG) and EY-6A (PDB ID, 6ZER) were shown in cartoon representation and colored in green, blue and magenta, respectively. **D**, Levels of anti-RBD antibodies at 10 μg/mL that block the cell (MDCK-spike-mCherry)-cell (MDCK-ACE2-GFP) fusion activity. Cells that undergo fusion form giant cells in yellow color. Images were acquired with original magnification 40x, scale bar 130 µm.

A panel of plasmablast-derived anti-RBD antibodies efficiently neutralized the SARS-CoV-2 pseudovirus and live virus 14 (Figure 1A) and targeted non-overlapping epitope groups (Figure S1). Three major epitope groups were defined by our panel of anti-RBD neutralizing antibodies: the head/neck, the left hip and the right hip epitope of the “squirrel” representation of the RBD 14 equivalent to the left shoulder, left flank and right shoulder epitopes in the “torso” RBD 11 (Figure 1C). FI-3A and FI-1C target the head and neck (left shoulder of the torso) of the RBD and are typical neutralizing antibodies that block the binding of SARS-CoV-2 RBD to ACE2 protein (Figure 1B). Both competed for binding to the RBD with each other and each substantially competed with REGN10933 10, C121 15 and mAb 384 11 for binding to the RBD (Figure S1 and Figure 1C).

FD-11A and FD-5D target the right hip of the RBD (right shoulder in the torso analogy), did not compete with REGN10933 and C121 but competed with REGN10987 10 and S309 16 for binding to the RBD (Figure S1 and Figure 1C). Both FD-11A and FD-5D partially inhibited the interaction of RBD with ACE2 through steric hindrance (Figure 1B).

We have previously determined the structure of another antibody of the set, EY-6A and discovered that this antibody binds to the RBD at the left hip (squirrel) 14 / left flank (torso) 11 epitope which is opposite to the REGN10987 and S309 binding sites 10, 16, 17 (Figure 1C) and does not interfere with the binding of ACE2 to RBD (Figure 1B). Thus EY-6A targets a distinct epitope on the RBD as compared to the other antibodies, which is further supported by its non-competitive binding to RBD with FI-3A, FI-1C, REGN10933, FD-11A, FD-5D and REGN10977 (Figure S1).

To further explore possible neutralization mechanisms of anti-RBD antibodies, we established a spike-mediated cell-cell fusion system to examine the fusion-inhibition activity of antibodies. We produced Madin-Darby canine kidney (MDCK)-human 2,6-sialyltransferase (SIAT1) cells that stably co-express SARS-CoV-2 spike protein and mCherry (MDCK-spike-mCherry) as effector cells, and MDCK-SIAT1 cells that stably co-express the human ACE2 and green fluorescent protein (GFP) reporter protein (MDCK-ACE2-GFP) as target cells. After effector cells and target cells were co-incubated overnight, syncytia were formed which appear yellow in color in the overlaid images of the GFP and Texas Red channels (Figure 1D). Figure 1D shows that FI-3A, FI-1C and FD-11A completely inhibited the cell-cell fusion and FD-5D and EY-6A exhibited partially inhibitory activities. By contrast, control antibody Z3B2 failed to inhibit the cell-cell fusion.

Taken together, functional data revealed that FI-3A, FI-1C and FD-11A interfere with the RBD-ACE2 interaction and prevent the spike protein-mediated cell-cell fusion, the latter function being also partly inhibited by FD-5D. This largely explains the substantial neutralizing potencies of these antibodies, with FI-3A and FD-11A being the most potent 14 (Figure 1 and Figure S2).

To elucidate the binding modes, detailed interactions with the spike and mechanism of neutralization, we determined a cryo-EM structure of FI-3A Fab with spike, and higher resolution crystal structures of three Fab complexes. These high-resolution structures were (i) FI-3A with RBD, (ii) FD-5D with RBD and (iii) a ternary complex of both FI-3A and FD-11A bound to RBD. The ability to generate the ternary complex is in line with previous findings that these two target non-overlapping epitopes on the viral RBD 14 (Figure 1C). For all of these structural studies the viral antigen was based on essentially the original Wuhan strain. Additionally, 2D class averages of particles from the Cryo-EM analysis of trimeric spike incubated for under 5 min with either FD-5D or FD-11A Fabs showed no evidence of trimeric spike. Incubation of FD-11A Fab with spike at a lower pH of 5.5 (slightly acidic pHs have been found to 'stabilize' trimeric spike in some cases) gave rise to distorted spike reconstructions of insufficient resolution for full interpretation. In the light of the crystal structures we can see no obvious reason why attachment of either of these Fabs should destabilize the spike. The successful analysis, of spike with FI-3A Fab, revealed a pre-fusion spike with one RBD in the 'up' configuration, atop of which was density consistent with the variable domains of a single bound Fab (Figure S3). The binding modes are identical between the cryo-EM and crystallographic structure determinations and further analysis therefore focusses on the higher resolution crystal structures.

When these structural results are taken together with the previously published complex structures of EY-6A 17 we find, as expected, that they bind at three distinct epitopes on the RBD.

### Binding of FI-1C

We were unable to obtain structural data on FI-1C, however we note that whilst the heavy chain variable gene usage, VH 3-11, is not very common, it matches that of mAb 384 identified as a potent neutralizer and competitor with ACE2 11. The two residues FL within heavy chain (HC) complementarity-determining region 3 (CDR3) which interact strongly with the RBD are also present in both (CARRSNRFLIAFDIW in FI-1C). However, unlike mAb 384, FI-1C retains binding activity with the E484K B.1.351 RBD mutation, despite conservation of residue Y50 of heavy chain CDR2, which in mAb 384 makes a hydrogen bond to residue E484 of the RBD, rendering it exquisitely sensitive to this mutation. Since the pattern of competition observed for FI-1C is consistent with a range of related binding modes, including that of 384 (Figure S1 and Figure 1C), we cannot accurately map its binding.

### Binding features of FI-3A

**Figure 2:**
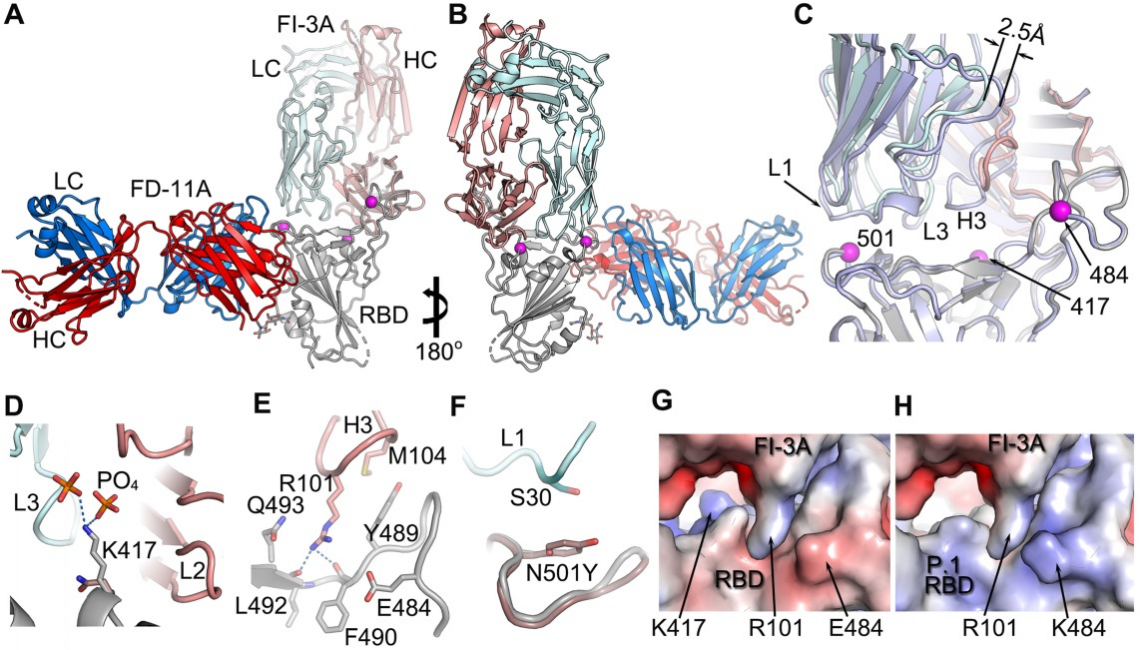
** The engagement of FI-3A on RBD. A**, The structure of RBD/FI-3A/FD-11A ternary complex. The RBD is in grey, the HC and LC of FD-11A, the HC and LC of FI-3A are in red, blue, salmon and pale cyan respectively. The mutation sites of the P.1 and B.1.351 variants are shown as magenta spheres. **B**, 180° rotation of **(A)**. **C**, comparing binding of FI-3A and COVOX-222 (HC grey, LC pale blue). **D**-**F**, Interactions between K417 of RBD and FI-3A **(D)**, RBD and H3 of FI-3A **(E)**, and N501 of the RBD and L1 of FI-3A **(F)**. The side chains of N471 and Y501 in the structure of B.1.351 variant RBD/EY-6A/COVOX-222 complex (PDB ID, 7NXA) are shown in **(D)** and **(F)** respectively. **G**, Electrostatic surface at the interface of RBD and FI-3A. **H**, the RBD of **(G)** is changed to the P.1 variant (PDB ID, 7NXC). HC, heavy chain; LC, light chain.

FI-3A belongs to the best studied class of public heavy chain V-regions, VH 3-53 mAb, and has a short heavy chain CDR3 (11 residues). These properties would lead us to expect it to bind at the back of the RBD neck/left shoulder 11, 14 (Figure 1C) in a very similar position and orientation to the numerous structurally available Fabs of this public antibody class (Figure 2 and Figure S4A, B). However, we find that some features of the binding of FI-3A are unique. Thus, after aligning the RBD of the FI-3A-Fab/RBD structure with the RBDs of four RBD/VH 3-53 Fab complexes we have studied previously (Fabs 150, 158, 222 and 269) 11 the HC CDR1 (H1) and HC CDR2 (H2) regions are well matched (RMSD < 1 Å), whilst the VL domain of FI-3A has an overall shift in both position and orientation, leading to a 2.5-4 Å displacement at the C-terminus of strand βC'' and a shift of 3-4.5Å at residue S30 of the L1 loop. such that S30 is more than 5 Å away from the side chain of RBD N501 11,18 (Figure S4A, B and Figure 2C). This will, unusually for a VH 3-53 antibody, render FI-3A resilient to mutation at residue 501, although an alternative mechanism of resilience has been observed for mAb 222 19.

### FD-5D and FD-11A bind at the same epitope

FD-5D and FD-11A represent one major class of anti-RBD neutralization antibodies, and compete with S309 and REGN10987 for RBD binding and bind RBD on the right hip 14 (Figure 1C and Figure S1).

**Figure 3:**
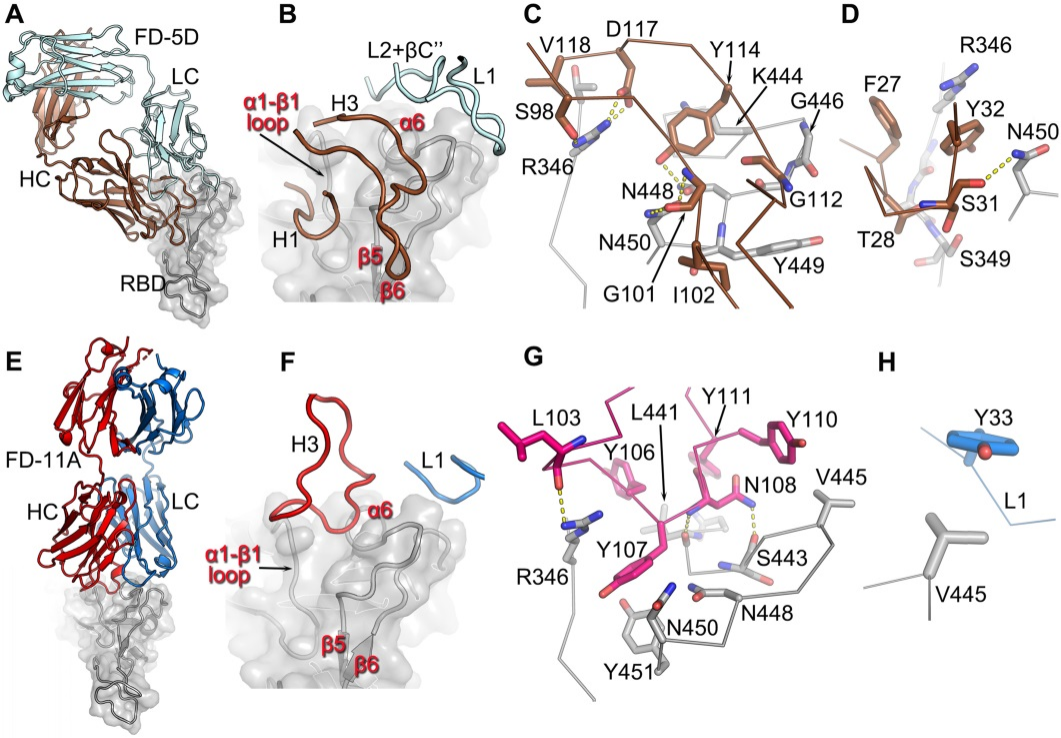
** The engagement of FD-5D and FD-11A on the RBD. A**, Overall structure of the RBD/FD-5D complex. The RBD is shown as a grey cartoon and semi-transparent surface. the FD-5D Fab is drawn with HC in brown and LC in pale cyan. **B**, CDR loops of FD-5D that have direct interactions (≤ 4 Å) with RDB. **C**,**D**, Details of interactions of FD-5D CDR H3 (**C**) and H1 (**D**) with the RBD. Side chains are shown as thicker sticks, and main chain backbone as thinner sticks. The color scheme is as in (**A**). Yellow broken bonds represent salt bridges and hydrogen bonds. **E**, Overall structure of FD-11A (HC red, LC blue) bound with the RBD (grey). **F**, Only CDRs H3 and L1 of FD-11A have direct contacts with the RBD. **G**,**H**, Detailed interactions of H3 (**G**) and L1 (**H**) of FD-11A with the RBD.

FD-5D makes a footprint of 880 Å^2^ on the RBD (670 Å^2^ and 211 Å^2^ from heavy and light chains respectively). Four CDRs, H1, H3 and Light Chain (LC) CDRs 1 and 2 (L1, L2) participate in the engagement with mainly α6-β5 loop (residues 444-452) and α1-β1 loop (residues 345-352) of the RBD (Figure 3A-D). The H3 loop is rather long (22 residues), but only the stem region (residues 97-102 and 111-118) is involved in binding, contributing a salt bridge from HC D117 to RBD R346 and three hydrogen bonds, two from HC G101 to RBD N450 and one from HC Y114 to RBD N448 (Figure 3C). The interactions of H1 are extensive and mainly comprise hydrophobic interactions with residues 347-349 of the α1-β1 loop and N450 from the α6-β5 loop of the RBD (Figure 3D).

Like FD-5D, FD-11A binds to the RBD right hip/shoulder 11, 14 but more on the side making a smaller footprint (680 Å^2^, 540 Å^2^ by heavy chain and 140 Å^2^ by light chain). Only two CDRs, H3 and L1, are involved in the interactions with the RBD, of which Y33 of L1 makes tight hydrophobic contacts with V445 of the RBD (Figure 3E-H). The 20 residue H3 loop engages with the α6-β5 and α1-β1 loops of the RBD (the loops that make extensive contacts with FD-5D H1). There are six tyrosine residues in the H3 loop, five of which are directly involved in the binding (Figure 3F, G; Y109 is not shown for clarity). RBD residues R346, N448 and N450 which make extensive interactions with FD-5D are also heavily involved in contacts with FD-11A. The FD-5D and FD-11A epitope, composed of α6-β5 and α1-β1 loops, is the same as that of REGN10987 10, although the mode of attachment and details of the interactions are different for the three antibodies. REGN10987 approaches the epitope in a similar direction to FD-11A but shifts about 8 Å to the back of the RBD such that none of the light chain residues are in contact with the RBD. REGN10987 engages the epitope using all three HC CDRs whereas FD-11A uses only H3 and L1, whilst FD-5D uses 4 CDRs, 2 from each chain (Figure S5).

### Simultaneous binding of antibody cocktail on the SARS-CoV-2 spike

**Figure 4:**
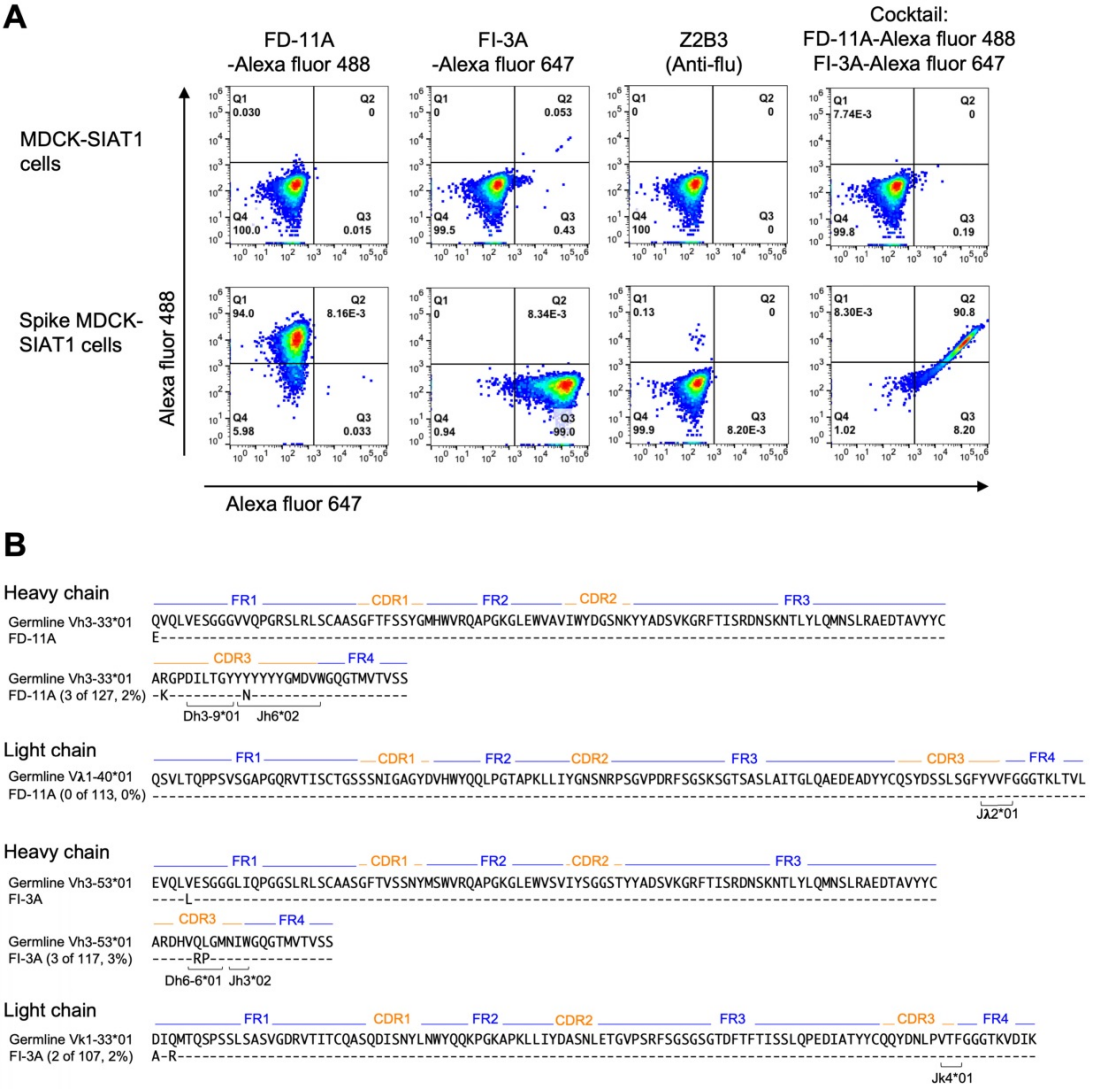
** Representative neutralizing anti-RBD antibodies FD-11A plus FI-3A as a cocktail. A**, The simultaneous binding of antibody cocktail FD-11A plus FI-3A with the spike protein expressed on the MDCK cell in the flow cytometry. Z2B3 is an anti-influenza neuraminidase human antibody. **B**, Immunogenetic analysis of the heavy and light chain variable regions of FD-11A and FI-3A using the international ImMunoGeneTics alignment tool. There are 4 and 0 somatic nucleotide mutations in heavy chain and light chain variable region genes in FD-11A, respectively. There are 4 and 3 somatic nucleotide mutations in heavy chain and light chain variable region genes in FI-3A, respectively. The numbers of amino acid substitutions in the heavy and light chain variable regions of FD-11A and FI-3A are shown in red in the figure.

The aim of selecting pairs of highly potent individual antibodies that could simultaneously bind the RBD spike was to identify ideal partners for an effective antibody cocktail that could also reduce the chance of escape mutant selection. The structural results showing simultaneous binding of FD-11A and FI-3A to the RBD were confirmed in the context of the cell-surface expressed full spike. FD-11A and FI-3A in a cocktail can bind simultaneously to the cell surface expressed full length spike equally strongly compared to when either are used alone (Figure 4A). The variable domain sequences of FD-11A and FI-3A were aligned with germline gene using the international ImMunoGeneTics alignment tool (http://www.imgt.org). Both antibodies are encoded with germline-like sequences with very low levels of somatic mutations (less than 4 nucleotide mutations) (Figure 4B), reinforcing that potently neutralizing antibodies should be readily elicited upon SARS-CoV-2 infection in humans.

### *In vivo* protection of antibody cocktail

**Figure 5:**
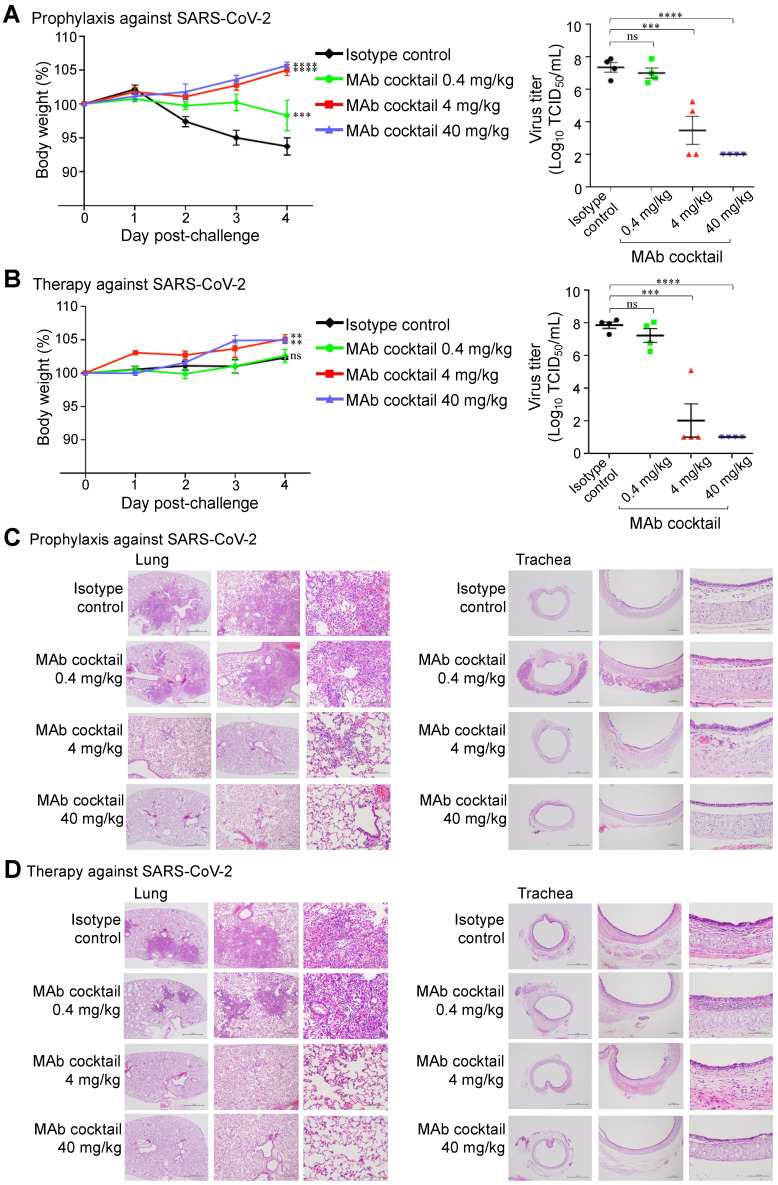
** The *in vivo* protection of FD-11A and FI-3A antibody cocktail against wild type SARS-CoV-2 in Syrian hamster model. A**, The prophylactic effect of antibody cocktail at 40 mg/kg, 4 mg/kg and 0.4 mg/kg. A single dose of antibody or control was administered intraperitoneally one day prior to intranasal challenge of virus.** B**, The therapeutic effect of antibody cocktail at 40 mg/kg, 4 mg/kg and 0.4 mg/kg. A single dose of antibody or isotype control was administered intraperitoneally three hours after intranasal challenge of virus. Body weight was measured at indicated time points and data were normalized to the initial weight of each animal. Infectious viral loads in the lungs were measured by median tissue culture infectious dose (TCID50) assay. The data represents the mean ± the standard error of the mean (SEM) (n=4 per group). Anti-influenza neuraminidase human IgG1 antibody Z2B3 was included as an isotype control. Statistical significance among groups was analyzed by two-way ANOVA and Tukey's post hoc test. The results of post hoc comparisons between the isotype control and treatment group were displayed in the graph. **, P < 0.01; ***, P < 0.001; ****, P < 0.0001; ns, not significant. **C**,**D**, Histopathological findings of the lungs in the** (C)** prophylactic and** (D)** therapeutic treatment of antibody cocktail at 40 mg/kg, 4 mg/kg and 0.4 mg/kg in hamsters four days after SARS-CoV-2 infection. H&E stain. 40x, 100x, 400x. Arrow.

We further evaluated the prophylactic and therapeutic efficacies of the FD-11A and FI-3A cocktail in the Syrian hamster model (Figure 5). In the prophylactic experiment, one day prior to challenge with SARS-CoV-2 (hCoV-19/Taiwan/4/2020), animals were treated with a single dose of FD-11A and FI-3A cocktail or an isotype control (Z3B2, anti-influenza hemagglutinin human IgG1 monoclonal antibody 20). Administration of antibody cocktail at 40 mg/kg (20 mg/kg of each antibody) or 4 mg/kg (2 mg/kg of each antibody) prior to SARS-CoV-2 challenge resulted in complete protection from weight loss (two-way ANOVA with post hoc tests, P < 0.0001) (Figure 5A). This protection was also accompanied by greatly decreased viral load in the lungs at the end of the study (day 4 post challenge) (two-way ANOVA with post hoc tests, P < 0.0001) (Figure 5A, Figure S6). We did note that a few treated animals with 4 mg/kg had substantial viral level in the lungs; however, these animals did not have significant weight loss compared to those with much lower viral loads. Administration of 0.4 mg/kg prevented a sharp decrease in body weight, but treated animals failed to gain weight at the end of study. Besides, we observed high viral loads in the lungs of 0.4 mg/kg antibody-treated animals.

In the therapeutic experiment, animals were treated with a single dose of FD-11A and FI-3A cocktail or isotype control three hours after challenge with SARS-CoV-2. We observed that animals treated with 40 and 4 mg/kg gradually gained weight and those treated with 0.4 mg/kg and isotype control had a significant reduction in weight gain (two-way ANOVA with post hoc tests, P < 0.01). The viral replication data demonstrated that animals treated with 40 or 4 mg/kg had low viral loads in the lungs (two-way ANOVA with post hoc tests, P < 0.0001) (Figure 5B, Figure S6). By contrast, animals treated with 0.4 mg/kg antibody or isotype control had similarly high viral loads in the lungs.

Animals were sacrificed on day 4 after viral challenge and the right lung and trachea were collected for histopathological evaluation. In the prophylactic experiment, there was a significantly lower amount of pulmonary inflammation or necrosis in animals treated with 40 or 4 mg/kg of antibody cocktail when compared to those treated with 0.4 mg/kg or isotype control (Figure 5C, Tables S1 and S2). Little pulmonary inflammation was found in animals treated with 40 mg/kg, and the level of inflammation was significantly lower when compared to those treated with 4 mg/kg. In addition, multifocal minimal to slight inflammation in the submucosa of the trachea was also seen. There was a significantly lower level of acute tracheal inflammation in animals treated with 40 or 4 mg/kg when compared to the 0.4 mg/kg or isotype control-treated group, and a complete recovery from inflammation was found in animals treated with 40 mg/kg. Similar histopathological findings of lung and trachea were observed in the therapeutic experiment (Figure 5D, Tables S1 and S2).

Taken together, the Syrian hamster study shows that the prophylactic or therapeutic treatment with either 40 or 4 mg/kg of antibody cocktail could significantly reduce lung viral load and attenuate SARS-COV-2 virus-induced pulmonary inflammation as judged by histopathological examination.

### The effect of viral variants on the function of anti-RBD antibodies

Several new variants have emerged since late 2020, i.e., the B.1.1.7, B.1.351, P.1, B.1.617.2 lineages, and G614 polymorphism. These variants, namely variants of concern, are worrying as they possess key mutations in the RBD which might escape neutralization of mAbs isolated from patients infected with the wild type SARS-CoV-2 virus. The B.1.1.7 lineage, also known as 20I/501Y.V1 and Alpha, was first identified in the United Kingdom in September 2020 and the spike protein contains characteristic mutations (e.g., N501Y within the RBD) compared with other circulating strains 21. The B.1.351 lineage, also known as 20H/501Y.V2 and Beta, was first identified in South Africa in late 2020 and arose independently to B.1.1.7 but shares several mutations, including the spike mutation N501Y. Moreover, the B.1.351 lineage contains two other mutations in the RBD (E484K and K417N) which have the potential to impact immunity from prior infection or vaccination 22-24. The P.1 lineage, also known as Gamma, was firstly identified in Brazil and then spread in the Americas and contains three mutations in the RBD (K417T, E484K, and N501Y) 19, 25. The B.1.617.2 lineage, also known as Delta, harbored L452R and T478K RBD mutations, was first detected in India in late 2020 and has become the most commonly reported variant since June 2021 26, 27. The G614 polymorphism has also been the dominant polymorphism globally over time 28.

**Figure 6:**
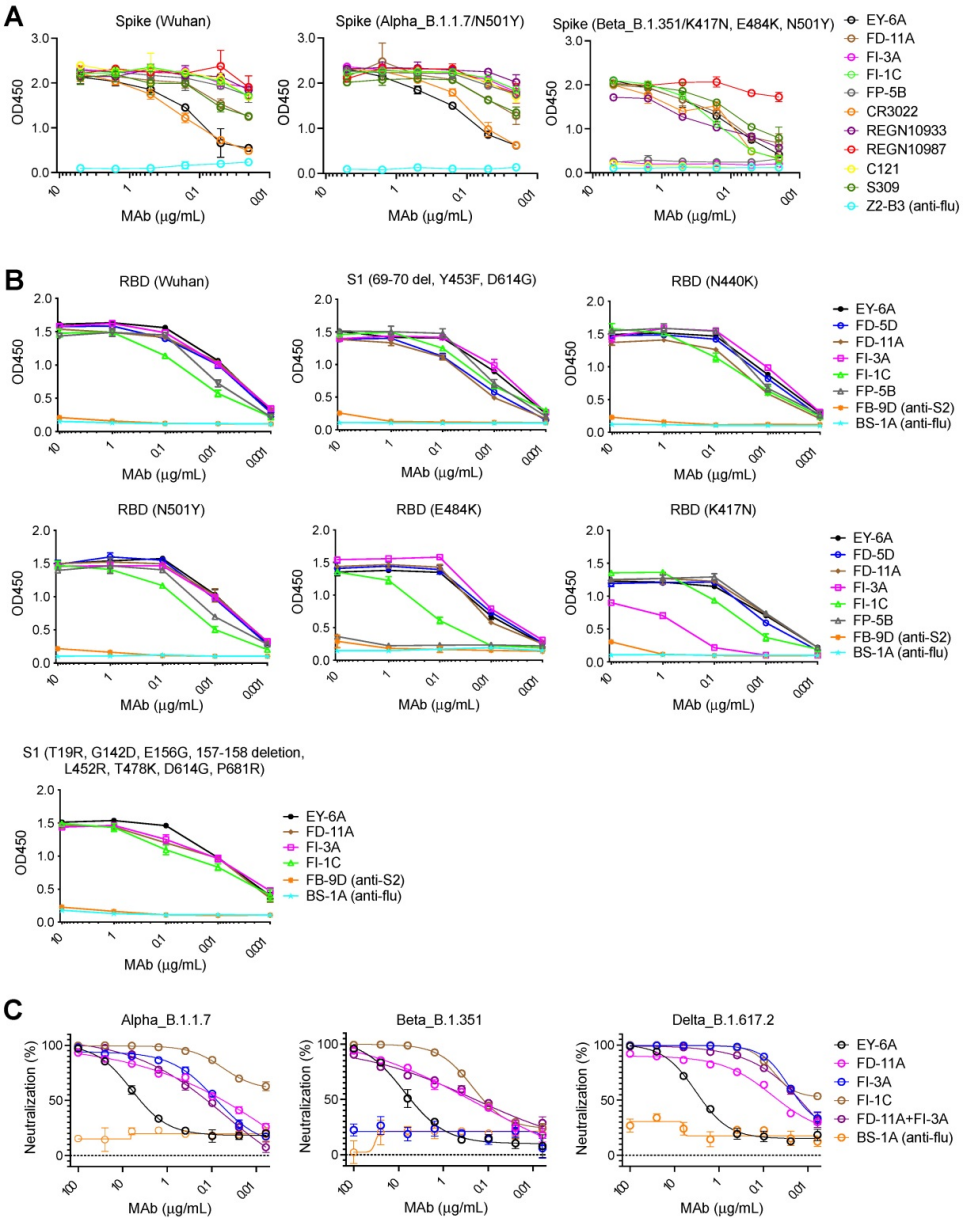
** The binding and neutralizing activities of anti-RBD human monoclonal antibodies with SARS-CoV-2 variants. A**, The binding of antibodies with full-length spikes of Alpha variant B.1.1.7 and Beta variant B.1.351 on the HexaPro backbone. The data represents the mean ± the standard error of mean with two replicates.** B**, The binding of antibodies on recombinant RBD proteins with single amino acid substitutions (i.e., N501Y, N440K, E484K or K417N) and recombinant S1 proteins with amino acid substitutions (Y453F, D614G and HV69-70 deletions; T19R, G142D, E156G, 157-158 deletion, L452R, T478K, D614G, and P681R). The data represents the mean ± the standard error of mean with two replicates. **C**, Neutralization of antibodies against SARS-CoV-2 Alpha, Beta and Delta pseudoviruses. BS-1A is an anti-influenza H3 human antibody and included as a control. The data represents the mean ± the standard error of mean with three replicates. The 50% inhibitory concentrations of EY-6A, FD-11A, FI-3A, FI-1C, FD-11A plus FI-3A against the Alpha variant B.1.1.7 were 3.704, 0.068, 0.082, 0.001 and 0.113 µg/mL, respectively. FI-3A fails to neutralize the Beta variant B.1.351 and the 50% inhibitory concentrations of EY-6A, FD-11A, FI-1C, FD-11A plus FI-3A against the Beta variant were 5.128, 0.201, 0.101 and 0.125 µg/mL, respectively. The 50% inhibitory concentrations of EY-6A, FD-11A, FI-3A, FI-1C, FD-11A plus FI-3A against the Delta variant B.1.617.2 were 2.114, 0.038, 0.014, 0.006 and 0.017 µg/mL, respectively.

Figure 6A shows that FD-11A and FI-3A strongly bind to the spike of B.1.1.7 lineage (Figure 6A) and neutralize the variant at a similar level to the wild type 26 (Figure 1A and Figure 6C). The spike N501Y and D614G mutations in the B.1.1.7 variant do not significantly alter the binding activity of the anti-RBD neutralizing antibodies tested (as described below, for FI-3A this in line with our structural observations) (Figure 6B). FD-11A binds to the spike of B.1.351 lineage in a similar fashion (Figure 6A) and effectively neutralizes the variant 26 (Figure 6C). This is exactly in line with the structural analysis which reveals that FD-11A does not have any direct contact to the mutation sites of B.1.1.7 and B.1.351 variants (Figure 3 and Figure S5), as is seen for REGN10987, which has also been shown to be resilient to these variants 11, 18, 24.

However, FI-3A has lost its binding activity with the spike of B.1.351 lineage (Figure 6A), which corresponds to the abrogation of neutralization with this emerging variant 26 (Figure 6C). Other antibodies FP-5B and C121 that recognize an overlapping epitope with FI-3A have also lost binding or neutralizing activity with the B.1.351 lineage 26 (Figure 6A). The characteristic mutation E484K in the B.1.351 lineage is responsible for the loss of binding of FP-5B (Figure 6B). By testing the binding on RBD with the individual lineage defining mutations of B.1.351 (E484K, K417N and N501Y), we show that FI-3A could tolerate E484K mutation but was sensitive to K417N (Figure 6B), which suggests that the K417N or the combination of K417N and E484K play a critical role in the escape of B.1.351 lineage from this antibody (Figure 2D-H). The underlying reason for this behavior is revealed by the structural analysis, which shows that FI-3A is an atypical VH 3-53 antibody, in that residue S30 of the light chain is displaced some 5 Å from the canonical position. Residue 30 is conserved as a serine in nearly all of the many VH 3-53 antibodies characterized and in antibodies 150, 158 and 269, which we have studied, has direct contacts (≤ 4 Å) with N501 in the RBD complex (Figure S4), such that steric clashes or altered interaction due to enlargement of the 501 side chain with the N501Y mutation compromises neutralization of these mAbs. In contrast a tyrosine at 501 of the RBD is unlikely to clash with the L1 loop of FI-3A, explaining its resistance to the N501Y mutation (Figure 2). We note that this is a different mechanism of conferring resilience to that previously reported for another VH 3-53 antibody, mAb 222, which instead uses a rare mutation to place a proline at VL residue 30 which can form stabilizing ring-stacking interactions with the tyrosine 11,19 (Figure 2C, F and Figure S4). However, in FI-3A, residue R101 of the CDR-H3 extends towards E484, making contacts to the side chains of Q493 and Y489, and hydrogen bonds to the carbonyl oxygens of F490 and L492 (Figure 2E). Although neither K417 nor E484 have any direct contact to the Fab, they contribute to the binding of FI-3A by providing charge complementarity (Figure 2H). Either K417N/T or E484K of the RBD will reduce the charge complementarity at the binding interface, impacting neutralization by FI-3A.

Neither FD-11A nor FI-3A makes direct contact with the mutation sites of the B.1.617.2 variant. The result also shows both antibodies retain their activities to bind the spike of the B.1.617.2 lineage and neutralize the B.1.617.2 variant (Figure 6B and Figure 6C).

Although the B.1.351 lineage develops resistance to FI-3A, the FD-11A plus FI-3A cocktail retained approximately equivalent neutralizing activity against circulating B.1.1.7 and B.1.351 variants (Figure S7 and Figure 6C). Interestingly, FI-1C retains binding and functional activity with the spike of the B.1.351 lineage (Figure 6A, Figure S8) 29. FI-1C also tolerates several characteristic RBD mutations in the B.1.1.7, B.1.351 and B.1.617.2 lineages in the binding assay, but a reduced binding with E484K was found (Figure 6B and Figure 6C). Since FI-1C showed neutralizing activity with the B.1.1.7, B.1.351 and B.1.617.2 variants (Figure S9, Figure 6C), FI-1C could be a promising alternative to use in a cocktail with FD-11A as a treatment option.

## Discussion

Our findings have relevance to the use of anti-RBD antibodies to treat or prevent SARS-CoV-2. The treatment of COVID-19 could be potentially achieved by combining potent neutralizing antibodies that target different epitopes 4, 7. Such cocktails (REGN10933 plus REGN10987, or LY-CoV555 plus LY-CoV016) have been authorized for emergency use for treatment of mild to moderate COVID-19 among certain subgroups of people. We identified and investigated an antibody cocktail (FI-3A plus FD-11A) that shares similar binding epitopes with the REGN10933 plus REGN10987 cocktail. As the COVID-19 outbreak is continuing to spread among the susceptible population, it has been observed that the virus accumulates mutations in its spike antigen and there is a risk that the virus escapes from the recognition of antibody-mediated neutralization 11, 18, 22, 26, 30. A longitudinal study demonstrated the isolation of the B.1.1.7 variant from respiratory samples and its reduced sensitivity to antibody in immunocompromised COVID-19 patients, supporting the emergence of antibody escape variants *in vivo* 31. Moreover, the mutation has been observed in multiple lineages and linked with several RBD mutations in the circulating SARS-CoV-2 variants 32. The E484K mutation in the B.1.351 variant compromises the activity of REGN10933 and LY-CoV555 32. The emergence of antigenic drift of SARS-CoV-2 highlights the importance of viral genomic surveillance and warrants proactive development of antibody therapeutics, including combinations that target antigenically distinct epitopes.

*In vitro* selection of escape mutants has been demonstrated by passaging virus expressing the SARS-CoV-2 spike protein in the presence of anti-RBD antibodies 30, 33, 34. Some approaches could be applied to curtail the selection of escape variants: a cocktail of non-overlapping anti-RBD antibodies or an anti-RBD plus anti-NTD antibody cocktail 14, 33-35. Neutralization by human antibodies to the NTD within the S1 domain has been detected 14, 35, but their mechanism of action is not known. The range of neutralizing activities is overlapping with those targeting the RBD. Cocktails of antibodies that include a representative binding to the NTD may reduce the likelihood of selecting neutralization-resistant viruses in the future.

Here we have analyzed the effectiveness of our candidate antibodies against variants B.1.1.7, B.1.351 and B.1.617.2. We find that whilst all are effective against B.1.1.7 and B.1.617.2, neutralization by some of the antibodies is abrogated by the changes in B.1.351. Interestingly the pattern of these effects is not what would be predicted from analysis of the broad epitopes and germline sequences, but is explained by the structural analysis which reveals, for instance for FI-3A, a significant modification of the canonical mode of engagement of VH 3-53 antibodies, conferring resilience to mutation at residue 501 of the RBD but sensitivity to mutations at 417 and 484.

In general neutralization of SARS-CoV-2 by human antibodies is tightly linked with the blockade of ACE2-RBD interaction, but there are some exceptions. For instance, we have shown previously that EY-6A binds the RBD strongly but does not interfere with ACE2 receptor attachment. EY-6A recognizes a cryptic and conserved RBD epitope which has a key role in stabilizing the pre-fusion form of the spike and the binding of antibody destabilizes the native spike conformation catalyzing conversion to the post-fusion form 14,17, the phenomenon is also found for a SARS-CoV and SARS-CoV-2 cross-reactive antibody, CR3022 36. EY-6A has potential as an immune-therapeutic particularly since it could be combined with antibodies blocking the ACE2 binding. A triple-antibody cocktail could be a reasonable option for clinical development as passive immunotherapy, since the production capacity of monoclonal antibodies is moving forward fast. To prove the concept of triple-antibody cocktail against SARS-CoV-2, we have demonstrated that simultaneous binding of FD-11A, FI-3A and EY-6A to the RBD and spike of virus expressed at the cell surface is feasible (Figure S10).

In this study, we assessed the *in vivo* prophylactic and treatment efficacy of the FD-11A plus FI-3A monoclonal antibody cocktail in Syrian hamsters. Our results demonstrate that the antibodies are efficacious, as measured by reduced viral load in the lung, by reduced virus-induced pathological inflammation, and by limited weight loss in the hamster model. The ability of 40 mg/kg antibody cocktail to substantially block detection of subgenomic RNA of SARS-CoV-2 (Figure S6) highlights the potential of this antibody cocktail to provide sterile immunity *in vivo*. This protective effect is similar to the protection afforded by the REGN10933 plus REGN10987 cocktail in another study 37, but a direct comparison of the activities of two RBD-targeted mAb cocktails was lacking. Furthermore, we did not observe any antibody-mediated enhancement evident by the absence of increased viral shedding or accelerated deterioration of lung inflammation in the presence of antibodies at either high or low doses in the hamster model. While ADE of disease is a general concern for the development of vaccines and antibody therapies, our results support the safety of current antibody-based interventions observed in the clinical studies 4, 6, 7, 38.

## Conclusions

In conclusion, we report fully human neutralizing antibodies that target non-overlapping epitopes on the RBD of SARS-CoV-2, functioning with mechanisms that involve inhibition of receptor attachment and the fusion process. Combination of potent FD-11A and FI-3A exhibits both prophylactic and therapeutic efficacy in a hamster model of SARS-CoV-2 infection and serves as a promising cocktail therapeutic.

## Methods

### Ethics statement

The study protocol and informed consent were approved by the Research and Ethics Committee at the Chang Gung Medical Foundation and the Taoyuan General Hospital, Ministry of Health and Welfare. All subjects provided signed informed consent. The study was carried out in accordance with the Declaration of Helsinki and good clinical practice guidelines.

### Monoclonal antibodies

Freshly separated peripheral blood mononuclear cells (PBMCs) or thawed PBMCs were stained with fluorescent-labelled antibodies to cell surface markers purchased from BD Biosciences, United States; Pacific blue anti-CD3, Fluorescein isothiocyanate anti-CD19, Phycoerythrin-Cy7 anti-CD27, Allophycocyanin-H7 anti-CD20, Phycoerythrin-Cy5 anti-CD38 and Phycoerythrin anti-IgG. The CD3^neg^CD19^pos^CD20^neg^CD27^hi^CD38^hi^IgG^pos^ cells were gated and isolated in chamber as single cells as previously described 14, 20. Heavy and light chain variable domains of single plasmablast cell were cloned into their respective vectors and expressed as human IgG1 as previously described 17.

### Pseudovirus neutralization assay

The pseudotyped lentivirus carrying SARS-CoV-2 Spike protein was generated by transiently transfecting human embryonic kidney (HEK) 293T cells with pCMV-ΔR8.91, pLAS2w.Fluc. Ppuro and different pcDNA3.1-nCoV-SΔ18 (wild type, Alpha, Beta or Delta variant).The 96-well plate was pre-seeded with 10,000 HEK 293T cells stably expressing human ACE2 gene at the time of infection. Pre-titrated SARS-CoV-2 pseudotyped lentivirus was mixed with the antibody preparation and incubated for 1 h at 37 °C. The virus-antibody mixture was then inoculated to target cells and infection was allowed to proceed for further 16 h at 37 °C and the culture medium was replaced with fresh Dulbecco's Modified Eagle Medium (DMEM) supplemented with 1% fetal bovine serum and 100 U/mL Penicillin/Streptomycin. After further 48 h of culture incubation, the Bright-Glo Luciferase Assay System (Promega, United States) was used to detect the luciferase activity and the Tecan Infinite F500 was used to detect the relative light unit. The virus control was set up in each assay. Antibody neutralization percentage was determined according to the relative light unit value as follows: [(relative light unit^Control^ - relative light unit^mAb^) / relative light unit^Control^] x 100%.

### Plaque reduction neutralization assay

In brief, this assay examines the concentration of antibody that produces a 50% reduction in numbers of authentic SARS-CoV-2 virus plaques in Vero E6 cells as previously described 17. Authentic SARS-CoV-2 (Australia/VIC01/2020, GenBank MT007544) 39 was diluted to an optimal concentration and mixed with doubling antibody dilutions and the mixture was incubated for 1 h at 37 °C. The virus-antibody mixture was then added into the wells of 24-well plate containing confluent monolayers of Vero E6 cells and incubated for 1 h at 37 °C in 5% CO2 incubator. The cell monolayer was then overlaid with MEM containing 1.5% carboxymethylcellulose (Sigma, United States), 4% (v/v) fetal bovine serum and 25 mM HEPES buffer. After further incubation for 5 days at 37 °C in 5% CO_2_ incubator, the plates were fixed with 20% formalin/PBS (v/v), stained with 0.2% crystal violet solution (Sigma, United States) and plaques were counted. A mid-point probit analysis (written in R programming language for statistical computing and graphics) was used to determine the dilution of antibody required to reduce numbers of SARS-CoV-2 virus plaques by 50% compared with the virus-only control.

### Microneutralization assay

In brief, this assay determines the concentration of antibody that produces a 50% reduction in infectious focus-forming units of authentic SARS-CoV-2 in Vero cells, as follows. Quadruplicate serial dilutions of serum or monoclonal antibody were preincubated with a fixed dose of SARS-CoV-2 before incubation with Vero cells. A 1.5% carboxymethyl cellulose-containing overlay was used to prevent satellite focus formation. Twenty hours post-infection, the monolayers were fixed with 4% paraformaldehyde, permeabilized with 2% Triton X-100 and stained for the nucleocapsid (N) antigen or spike (S) antigen using monoclonal antibody EY-2A and EY-6A 14, respectively. After development with a peroxidase-conjugated antibody and TrueBlue peroxidase substrate, infectious foci were enumerated by enzyme-linked immune absorbent spot reader. Data were analyzed using four-parameter logistic regression (Hill equation) in GraphPad Prism 8.3.

### Competitive binding assay

Competitive binding assays were performed as described previously 14. In brief, the NUNC plate was pre-coated with RBD-VLP 40 overnight at 4ºC. After the plate was washed and blocked with 5% dried skimmed milk in PBS for 1 h at room temperature, the mixture of biotinylated antibody (EZ-Link Sulfo-NHS-LC-biotin, Life Technologies, United States) and at least ten-fold excess of competing antibody was added to the plate and incubated for 1 h. After washing, the plate was incubated with Streptavidin-Horseradish Peroxidase conjugate (Life Technologies, United States) for another one hour. After the plate was washed, the signal was developed using the POD substrate (Roche, Switzerland) and the reaction was stopped with 1M H_2_SO_4_. The OD450 value was measured using a Clariostar plate reader (BMG Labtech, Germany). The mean and 95% confidence interval of four replicates were calculated. Competition was measured as: (X-minimum binding/(maximum binding-minimum binding), where X is the binding of the biotinylated antibody in the presence of competing antibody. Minimum binding is the self-blocking of the biotinylated antibody or background binding. Maximum binding is binding of biotinylated antibody in the presence of non-competing antibody (anti-influenza neuraminidase antibody Z3B2).

### ACE2-blocking assay

MDCK-ACE2 cells were produced by stably transfecting parental MDCK-SIAT1 cells to overexpress codon-optimized human ACE2 cDNA (NM_021804.1) using lentiviral vector and then FACS sorted. MDCK-ACE2 cells were prepared and resuspended. The RBD with a BirA tag was biotinylated using the biotinylation kit (Avidity LLC, United States). Pre-titrated biotinylated RBD was mixed with serial dilutions of the antibody and the mixture was incubated with MDCK-ACE2 cells for 1 h. After washing, the cells were incubated with ExtrAvidin-R-Phycoerythrin (Sigma, United States) for 30 min at room temperature. The frequency of RBD-bound cells was analyzed by BD FACSCanto II flow cytometer (BD Biosciences, United States). At least 2,500 cells were collected for analysis. The mean and 95% confidence interval of two replicates were calculated. The PBS-biotinylated RBD mixture was used to obtain maximum signal and PBS only was used to determine background. The relative inhibition rates of each dilution of sample were calculated according to the frequency as follows: inhibition rate = [1 - (average RBD-bound cell frequency of sample - average RBD-bound cell frequency of PBS only control)/(average RBD-bound cell frequency of maximal signal control - average RBD-bound cell frequency of PBS only control)] × 100%.

### Production of monoclonal antibodies

The expression plasmids for FI-3A, FD-11A, FD-5D, EY-6A and FI-1C were created as previously described 14. The VH and VL genes of C121 15, REGN10933 10,33, REGN10987 10,33 and S309 16 were synthesized as cDNA fragments and subcloned into the AbVec Heavy, AbVec Kappa or AbVec Lambda vector as previously described 14. Control mAb Z2-B3 is an anti-influenza neuraminidase mAb that was described previously 20. Monoclonal antibodies were produced in ExpiCHO cell line according to manufacturer's instructions and affinity purified using a MabSelect SuRe (Cytiva) pre-packed column. Purified mAbs were then desalted using Zeba Spin Desalting Column (ThermoFisher) or a PD-10 Desalting Column (GE Healthcare).

### Cell-Cell fusion assay

The cell lines used in the fusion assay were produced by stably transfecting MDCK-SIAT1 (ECACC 05071502) using a lentiviral vector as previously described 17. In brief, MDCK-ACE2-GFP cells were created by stably transfecting with lentiviral vectors carrying a codon-optimized human ACE2 cDNA transgene or enhanced GFP cDNA transgene, respectively. MDCK-Spike-mCherry were created by stably transfecting with lentiviral vector carrying a codon-optimized SARS-CoV-2 Spike (Wuhan strain) cDNA transgene or mCherry reporter protein cDNA transgene, respectively. MDCK-ACE2-GFP and MDCK-Spike-mCherry cells were then sorted using a FACS Aria cell sorter. MDCK-ACE2-GFP cells were stained with purified SARS-CoV-2 RBD-His followed by anti-6x-His Tag Alexa Fluor 647 mAb (ThermoFisher MA1-15-A647) and double positive cells (AF647 and GFP) were sorted and expanded. MDCK-Spike-mCherry cells were stained with purified anti-RBD mAb EY-6A followed by Goat Anti-Human Alexa Fluor 488 mAb (ThermoFisher A48276) and double positive cells (AF488 and mCherry) were sorted and expanded. To run the fusion assay, monoclonal antibodies diluted in 100 μL (20 μg/mL) of D10 (DMEM, 10% fetal calf serum, penicillin (100 U/mL) and streptomycin (100 μg/mL)) were added to a well (in duplicate) on a flat-bottomed 96-well plate. 50 μL of MDCK-Spike-mCherry (300,000 cells/mL) and 50 μL of MDCK-ACE2-GFP (300,000 cells/mL) cells were then added and the plates were incubated overnight at 37 °C. The media was then removed and the cells were fixed with 100 μL 10% formalin at 4 °C for 30 m. The cells were then visualized under a fluorescent microscope (Revolve, ECHO) and the images of each well on the GFP and Texas Red channel were captured and merged.

### Hemagglutination assay for detection of antibodies to the RBD

The hemagglutination assay was performed as described 29. Briefly, doubling dilutions of monoclonal antibodies (from 20 μg/mL in PBS stock) were prepared in 50 μL PBS in V bottomed microtiter plates. Red cells (50 μL of 1:40 dilution in PBS whole blood from a seronegative donor) were added. Finally, 50 μL of the developing reagent of IH4-RBD (2 μg/mL stock solution) was added. The IH4 nanobody to Glycophorin A binds the RBD to the red cell surface. The RBD sequence was adjusted by site directed mutagenesis to match each variant of concern. Control negative wells contained PBS but no mAb. After 1 h the plates were tilted for ~20 s and photographed. Negative wells showed any flow of the red cell button as a teardrop, and positive wells showed complete absence of teardrop (Figure S8).

### Production of soluble HexaPro Spike proteins and Enzyme-linked immunosorbent assays (HexaPro Spike)

Trimeric SARS-CoV-2 Spike (HexaPro) (Wuhan) was expressed in Expi293 cells according to the manufacturer's instructions using the pαh-SARS-CoV-2 HexaPro Spike plasmid obtained from Addgene (154754). The Trimeric SARS-CoV-2 Spike (HexaPro) (Wuhan) was then affinity purified using a HisTrap HP (GE Healthcare 17-5247-01) and desalted using a Zeba Spin Desalting Column, 7K MWCO (ThermoFisher 89893). cDNA encoding trimeric SARS-CoV-2 Spike for B.1.1.7 (Alpha) and B.1.351 (Beta) variants flanked by KpnI and XhoI restriction sites were synthesized as codon optimized fragments (Integrated DNA Technologies) and subcloned into the pαh vector. Trimeric SARS-CoV-2 Spike B.1.1.7 (Alpha) and B.1.351 (Beta) were expressed and purified as described above. For the enzyme-linked immunosorbent assay, NUNC plates were coated with 1 μg/mL of affinity purified HexaPro Spike proteins in PBS and incubated at room temperature for 2 h. The plates were then blocked with 5% skim milk in PBS overnight at 4 °C. After removing the blocking buffer and washing three times with PBS, the plate was incubated with serial dilutions of the antibody (diluted in PBS/0.1% bovine serum albumin) for 60 min at room temperature. After washing three times with PBS, the plate was incubated with horseradish peroxidase-conjugated rabbit anti-human IgG (1:1,600) (Agilent, P0214020-2) in PBS/0.1% bovine serum albumin for 1 h at room temperature. After washing three times with PBS, the plate was developed with BM Blue POD substrate (Roche, 11484281001) for 5 min at room temperature before stopping with 1M sulfuric acid. The absorbance was measured at OD450 on a Clariostar microplate reader (BMG Labtech).

### Enzyme-linked immunosorbent assays (RBD)

The NUNC plate was coated with recombinant RBD proteins (Sino Biological, China) in carbonate buffer and incubated at 4 °C for 16 h. Nonspecific binding was blocked with the solution of 3% bovine serum albumin for 1 h at room temperature on a shaker. After removing the blocking buffer, the plate was incubated with serial dilutions of the antibody for 60 m at 37 °C. After washing, the plate was incubated with horseradish peroxidase-conjugated anti-human or anti-mouse IgG (Rockland Immunochemicals, United States) for 60 min at room temperature. After washing, the plate was developed with TMB substrate reagent (BD Biosciences, United States). The reaction was stopped by 0.5M hydrochloric acid and the optical density was measured at OD450 on a microplate reader.

### Cloning of RBD

The construct of RBD used in this study is the same as previously described 17. DNA of RBD was amplified by PCR, digested with restriction enzymes AgeI and KpnI and then ligated with digested pNEO vector.

### Protein production

Protein expression and purification were performed as described previously 17. Briefly RBD and mAbs were expressed in HEK 293T cells. The mAbs were purified by protein-A and His-tagged RBD by Ni-NTA and gel filtration.

### Preparation of Fab fragments

Fab fragments of FD-5D, FD-11A and FI-3A antibodies were digested and purified using a Pierce Fab Preparation Kit, following the manufacturer's protocol.

### Crystallization

FD-5D and FI-3A Fabs were mixed with RBD separately with a final concentration of 13 mg/mL. FI-3A and FD-11A Fabs were mixed with RBD in a 1:1:1 molar ratio with a final concentration of 7 mg/mL. After incubation at room temperature for 30 min, the samples were used for initial screening of crystals in Crystalquick 96-well X plates (Greiner Bio-One) with a Cartesian Robot using the nanoliter sitting-drop vapor-diffusion method with 100 nL of protein and 100 nL of reservoir in each drop, as previously described 18. Crystals of RBD/FD-5D Fab complex were obtained from Molecular Dimensions Morpheus screen, condition C9 containing 10% (w/v) PEG 20 000, 20% (v/v) PEG MME 550, 0.03 M of each NPS (NaNO_3_; Na_2_HPO_4_; (NH_4_)_2_SO_4_) and 0.1 M bicine/Trizma base pH 8.5. Tiny Crystals of RBD/FI-3A Fab complex were first formed in Hampton Research PEGRx screen, condition A10, containing 0.1 M sodium citrate tribasic and 30% (w/v) PEG 550 and further optimized in 0.08 M sodium citrate tribasic and 24% (w/v) PEG 550. Crystals of RBD/FI-3A/FD-11A Fabs complex were formed in Hampton Research Index screen, condition 25, containing 3.5 M sodium formate pH 7.0 and further optimized in 2.9 M sodium formate pH 7.0.

### X-ray data collection, structure determination and refinement

Crystals were mounted in loops and dipped in a solution containing 25% glycerol and 75% mother liquor for 1 s before being frozen in liquid nitrogen prior to data collection. Diffraction images of 0.1° rotation were recorded on an Eiger2 XE 16M detector, exposure time 0.006 to 0.009 s per image, beam size 80×20 μm. Diffraction data were collected at 100 K at beamline I03, 100% beam transmission and wavelength of 0.9763 Å). 360° of data was collected from each crystal. Data were indexed, integrated and scaled with the automated data processing program Xia2-dials 41, 42. A data set for each of the RBD/FD-5D and RBD/FI-3A complexes was merged from 3 crystals and 5 crystals for the ternary complex RBD/FI-3A/FD-11A.

Structures of the complexes were determined by molecular replacement with PHASER 43 using the RBD, VHVL and CHCL domains of SARS-CoV-2 RBD-EY6A-H4 (PDB ID 6ZCZ) 17 and RBD-158 (PDB ID, 7BEK) 11 complexes as search models. Model rebuilding with COOT 44 and refinement with PHENIX 45 were done for all the structures. Data collection and structure refinement statistics are given in Table S3. Structural comparisons used SHP 46, residues forming the RBD/Fab interface were identified with PISA 47 and figures were prepared with PyMOL (The PyMOL Molecular Graphics System, Version 1.2r3pre, Schrödinger, LLC).

### Cryo-electron microscopy studies

Recombinant spike ectodomain with a mutated furin site, double proline stabilizing mutations and a foldon C-terminal motif 17, at pH 4.6 was mixed with FI-3A at a 6-fold molar excess of trimeric spike. Each grid was prepared using 3 μL sample applied to a freshly glow-discharged (Plasma Cleaner PDC-002-CE, Harrick Plasma set to high, 20 s) holey carbon-coated 200-mesh copper grid (C-Flat, CF-2/1, Protochips) and excess liquid removed by blotting for 5-5.5 s with a blotting force of -1 using vitrobot filter paper (grade 595, Ted Pella Inc.) at 4.5 ºC, 100% relative humidity. Blotted grids were then immediately plunge frozen using a Vitrobot Mark IV (ThermoFisher). All grids were initially screened on a Glacios microscope operating at 200 kV (ThermoFisher). Data were collected on a Krios microscope (ThermoFisher), with a K2 detector (Gatan) at a magnification of 165 kX using SerialEM. Alignment and motion correction was performed using Cryosparc 48. Images were then manually inspected and those with poor CTF-fits were discarded. Particles were then picked by unbiased blob picking in Cryosparc v.2.14.1 and subjected to two rounds of 2D classification before three ab initio volumes were generated for unbiased heterogeneous refinement. A single class with a good orientational distribution with trimeric spike in a 1-RBD up configuration was then selected and refined using the non-uniform refinement module. Very weak density consistent with a fab decorating an RBD in the up position. From this initial particle set of 21600 particles, yielding a structure of 3.6 Å resolution, further 3D classification and local refinement procedures were conducted to try to better resolve the Fab/RBD interface. Following 3D classification, 9095 particles were refined to a final reported resolution of 4.8 Å. For local refinement procedures, the best strategy involved first subtracting density corresponding to the S2 and NTD regions of Spike from the first reconstructed volume, followed by 'legacy' local refinement with a mask around the RBD/fab region in Cryosparc. This was then used in a second round of local refinement using the latest Local Refinement module in Cryosparc, resulting in a map at 6.2 Å reported resolution that showed a slight improvement at the RBD up/fab interface (Figure S3). For details see Table S4.

A combination of spike ectodomain with RBD in the up position (PDB 7ND5) and the crystal structure of RBD with FI-3A (variable domains, 1-117, 1-108 for the H and L chains, respectively) were initially manually fitted and combined using Chimera1.14 (http://www.cgl.ucsf.edu/chimera) 49 before three cycles of rigid body refinement with NCS constraints and the remaining settings set to default in Phenix1.19.1-4122 45. The overall low correlation coefficient is due to the relatively poor map around the fab region (CC for the Spike ectodomain alone was 0.7). Resulting statistics are displayed in Table S4.

### Animal studies

Animal experiments were performed in accordance with the protocol approved by the Institutional Animal Care and Use Committee in the National Health Research Institutes, Taiwan. The experiments were carried out in accordance with the 'Guide for the care and use of laboratory animals', the recommendations of the Institute for Laboratory Animal Research, and Association for Assessment and Accreditation of Laboratory Animal Care International standards.

A total of 32 Syrian hamsters, female, 8 weeks old, were randomly divided into 8 groups (n=4 per groups) for the prophylactic or therapeutic studies. Animals were weighed prior to the start of the study to determine the baseline. Body weights were measured once daily during the study period. Antibodies were administered through intraperitoneal injection. Animals were challenged with 1×10^5 TCID_50_ of SARS-CoV-2 (hCoV-19/Taiwan/4/2020) by intranasal administration of 0.05 mL of viral inoculum into each nostril. Hamsters were sacrificed on day four after viral challenge and the right lobe of the lungs and trachea were collected for histopathological evaluation. Left lung tissues were harvested for viral load assays. Viral titers were determined by the TCID_50_ assay based on endpoint dilution and by the quantitative reverse transcription PCR (RT-PCR) for detection of both the SARS-CoV-2 envelope and nucleocapsid genes. The following primers and probes for the RT-PCR were used: Envelop gene_Sarbeco_F 5'-ACAGGTACGTTAATAGTTAATAGCGT-3', Envelop gene_Sarbeco _R 5'-ATATTGCAGCAGTACGCACACA-3', Envelop gene_Sarbeco _Probe FAM-ACACTAGCCATCCTTACTGCGCTTCG-BHQ1, CCDC-Nucleocapsid gene-F 5'-GGGGAACTTCTCCTGCTAGAAT-3', CCDC-Nucleocapsid gene-R 5'-CAGACATTTTGCTCTCAAGCTG-3', CCDC-Nucleocapsid gene-Probe HEX-TTGCTGCTGCTTGACAGATT-BHQ1.

## Supplementary Material

Supplementary figures and tables.

## Abbreviations

RBD: receptor-binding domain
SARS-CoV-2: severe acute respiratory syndrome coronavirus 2
ACE2: angiotensin-converting enzyme 2
mAb: monoclonal antibodies
MDCK: Madin-Darby canine kidney
SIAT1: human 2,6-sialyltransferase
GFP: green fluorescent protein
VH: heavy chain variable region
CDR: complementarity-determining region
VL: light chain variable region
HC: heavy chain
LC: light chain
PBMCs: peripheral blood mononuclear cells
DMEM: Dulbecco's Modified Eagle Medium

## References

[1] Zhu N, Zhang D, Wang W, Li X, Yang B, Song J (2020). A Novel Coronavirus from Patients with Pneumonia in China, 2019. N Engl J Med.

[2] Coronaviridae Study Group of the International Committee on Taxonomy of V (2020). The species severe acute respiratory syndrome-related coronavirus: classifying 2019-nCoV and naming it SARS-CoV-2. Nat Microbiol.

[3] Creech CB, Walker SC, Samuels RJ (2021). SARS-CoV-2 Vaccines. JAMA.

[4] Gottlieb RL, Nirula A, Chen P, Boscia J, Heller B, Morris J (2021). Effect of Bamlanivimab as Monotherapy or in Combination With Etesevimab on Viral Load in Patients With Mild to Moderate COVID-19: A Randomized Clinical Trial. JAMA.

[5] Katz LM (2021). (A Little) Clarity on Convalescent Plasma for Covid-19. N Engl J Med.

[6] Libster R, Perez Marc G, Wappner D, Coviello S, Bianchi A, Braem V (2021). Early High-Titer Plasma Therapy to Prevent Severe Covid-19 in Older Adults. N Engl J Med.

[7] Weinreich DM, Sivapalasingam S, Norton T, Ali S, Gao H, Bhore R (2021). REGN-COV2, a Neutralizing Antibody Cocktail, in Outpatients with Covid-19. N Engl J Med.

[8] Lan J, Ge J, Yu J, Shan S, Zhou H, Fan S (2020). Structure of the SARS-CoV-2 spike receptor-binding domain bound to the ACE2 receptor. Nature.

[9] Walls AC, Park YJ, Tortorici MA, Wall A, McGuire AT, Veesler D (2020). Structure, Function, and Antigenicity of the SARS-CoV-2 Spike Glycoprotein. Cell.

[10] Hansen J, Baum A, Pascal KE, Russo V, Giordano S, Wloga E (2020). Studies in humanized mice and convalescent humans yield a SARS-CoV-2 antibody cocktail. Science.

[11] Dejnirattisai W, Zhou D, Ginn HM, Duyvesteyn HME, Supasa P, Case JB (2021). The antigenic anatomy of SARS-CoV-2 receptor binding domain. Cell.

[12] Modjarrad K (2016). Treatment strategies for Middle East respiratory syndrome coronavirus. J Virus Erad.

[13] Group PIW, Multi-National PIIST, Davey RT Jr, Dodd L, Proschan MA, Neaton J (2016). A Randomized, Controlled Trial of ZMapp for Ebola Virus Infection. N Engl J Med.

[14] Huang KA, Tan TK, Chen TH, Huang CG, Harvey R, Hussain S (2021). Breadth and function of antibody response to acute SARS-CoV-2 infection in humans. PLoS Pathog.

[15] Robbiani DF, Gaebler C, Muecksch F, Lorenzi JCC, Wang Z, Cho A (2020). Convergent antibody responses to SARS-CoV-2 in convalescent individuals. Nature.

[16] Pinto D, Park YJ, Beltramello M, Walls AC, Tortorici MA, Bianchi S (2020). Cross-neutralization of SARS-CoV-2 by a human monoclonal SARS-CoV antibody. Nature.

[17] Zhou D, Duyvesteyn HME, Chen CP, Huang CG, Chen TH, Shih SR (2020). Structural basis for the neutralization of SARS-CoV-2 by an antibody from a convalescent patient. Nat Struct Mol Biol.

[18] Supasa P, Zhou D, Dejnirattisai W, Liu C, Mentzer AJ, Ginn HM (2021). Reduced neutralization of SARS-CoV-2 B.1.1.7 variant by convalescent and vaccine sera. Cell.

[19] Dejnirattisai W, Zhou D, Supasa P, Liu C, Mentzer AJ, Ginn HM (2021). Antibody evasion by the P.1 strain of SARS-CoV-2. Cell.

[20] Huang KA, Rijal P, Jiang H, Wang B, Schimanski L, Dong T (2019). Structure-function analysis of neutralizing antibodies to H7N9 influenza from naturally infected humans. Nat Microbiol.

[21] Muik A, Wallisch AK, Sanger B, Swanson KA, Muhl J, Chen W (2021). Neutralization of SARS-CoV-2 lineage B.1.1.7 pseudovirus by BNT162b2 vaccine-elicited human sera. Science.

[22] Tegally H, Wilkinson E, Giovanetti M, Iranzadeh A, Fonseca V, Giandhari J (2021). Detection of a SARS-CoV-2 variant of concern in South Africa. Nature.

[23] Wibmer CK, Ayres F, Hermanus T, Madzivhandila M, Kgagudi P, Oosthuysen B (2021). SARS-CoV-2 501Y.V2 escapes neutralization by South African COVID-19 donor plasma. Nat Med.

[24] Zhou D, Dejnirattisai W, Supasa P, Liu C, Mentzer AJ, Ginn HM (2021). Evidence of escape of SARS-CoV-2 variant B.1.351 from natural and vaccine-induced sera. Cell.

[25] Faria NR, Mellan TA, Whittaker C, Claro IM, Candido DDS, Mishra S (2021). Genomics and epidemiology of the P.1 SARS-CoV-2 lineage in Manaus, Brazil. Science.

[26] Skelly DT, Harding AC, Gilbert-Jaramillo J, Knight ML, Longet S, Brown A (2021). Two doses of SARS-CoV-2 vaccination induce robust immune responses to emerging SARS-CoV-2 variants of concern. Nat Commun.

[28] Plante JA, Liu Y, Liu J, Xia H, Johnson BA, Lokugamage KG (2021). Spike mutation D614G alters SARS-CoV-2 fitness. Nature.

[29] Townsend A, Rijal P, Xiao J, Tan TK, Huang KA, Schimanski L (2021). A haemagglutination test for rapid detection of antibodies to SARS-CoV-2. Nat Commun.

[30] Starr TN, Greaney AJ, Addetia A, Hannon WW, Choudhary MC, Dingens AS (2021). Prospective mapping of viral mutations that escape antibodies used to treat COVID-19. Science.

[31] Kemp SA, Collier DA, Datir RP, Ferreira I, Gayed S, Jahun A (2021). SARS-CoV-2 evolution during treatment of chronic infection. Nature.

[32] Wang P, Nair MS, Liu L, Iketani S, Luo Y, Guo Y (2021). Antibody resistance of SARS-CoV-2 variants B.1.351 and B.1.1.7. Nature.

[33] Baum A, Fulton BO, Wloga E, Copin R, Pascal KE, Russo V (2020). Antibody cocktail to SARS-CoV-2 spike protein prevents rapid mutational escape seen with individual antibodies. Science.

[34] Weisblum Y, Schmidt F, Zhang F, DaSilva J, Poston D, Lorenzi JC (2020). Escape from neutralizing antibodies by SARS-CoV-2 spike protein variants. Elife.

[35] Suryadevara N, Shrihari S, Gilchuk P, VanBlargan LA, Binshtein E, Zost SJ (2021). Neutralizing and protective human monoclonal antibodies recognizing the N-terminal domain of the SARS-CoV-2 spike protein. Cell.

[36] Huo J, Zhao Y, Ren J, Zhou D, Duyvesteyn HME, Ginn HM (2020). Neutralization of SARS-CoV-2 by Destruction of the Prefusion Spike. Cell Host Microbe.

[37] Baum A, Ajithdoss D, Copin R, Zhou A, Lanza K, Negron N (2020). REGN-COV2 antibodies prevent and treat SARS-CoV-2 infection in rhesus macaques and hamsters. Science.

[38] Li L, Zhang W, Hu Y, Tong X, Zheng S, Yang J (2020). Effect of Convalescent Plasma Therapy on Time to Clinical Improvement in Patients With Severe and Life-threatening COVID-19: A Randomized Clinical Trial. JAMA.

[39] Caly L, Druce J, Roberts J, Bond K, Tran T, Kostecki R (2020). Isolation and rapid sharing of the 2019 novel coronavirus (SARS-CoV-2) from the first patient diagnosed with COVID-19 in Australia. Med J Aust.

[40] Tan TK, Rijal P, Rahikainen R, Keeble AH, Schimanski L, Hussain S (2021). A COVID-19 vaccine candidate using SpyCatcher multimerization of the SARS-CoV-2 spike protein receptor-binding domain induces potent neutralising antibody responses. Nat Commun.

[41] Winter G (2010). xia2: an expert system for macromolecular crystallography data reduction. J Appl Cryst.

[42] Winter G, Waterman DG, Parkhurst JM, Brewster AS, Gildea RJ, Gerstel M (2018). DIALS: implementation and evaluation of a new integration package. Acta Crystallogr D Struct Biol.

[43] McCoy AJ, Grosse-Kunstleve RW, Adams PD, Winn MD, Storoni LC, Read RJ (2007). Phaser crystallographic software. J Appl Crystallogr.

[44] Emsley P, Cowtan K (2004). Coot: model-building tools for molecular graphics. Acta Crystallogr D Biol Crystallogr.

[45] Liebschner D, Afonine PV, Baker ML, Bunkoczi G, Chen VB, Croll TI (2019). Macromolecular structure determination using X-rays, neutrons and electrons: recent developments in Phenix. Acta Crystallogr D Struct Biol.

[46] Stuart DI, Levine M, Muirhead H, Stammers DK (1979). Crystal structure of cat muscle pyruvate kinase at a resolution of 2.6 A. J Mol Biol.

[47] Krissinel E, Henrick K (2007). Inference of macromolecular assemblies from crystalline state. J Mol Biol.

[48] Punjani A, Rubinstein JL, Fleet DJ, Brubaker MA (2017). cryoSPARC: algorithms for rapid unsupervised cryo-EM structure determination. Nat Methods.

[49] Pettersen EF, Goddard TD, Huang CC, Couch GS, Greenblatt DM, Meng EC (2004). UCSF Chimera-a visualization system for exploratory research and analysis. J Comput Chem.

