# Supplementary material for: Structures and therapeutic potential of anti-RBD human monoclonal antibodies against SARS-CoV-2
Source: Theranostics. 2022 Jan 1;12(1):1–17. doi: 10.7150/thno.65563 (PMC8690919; doi:10.7150/thno.65563)
Supplement: Supplementary file 1 — Supplementary figures and tables. [file thnov12p0001s1.pdf]

Table S1. Summary of pathological incidence of the lungs and trachea in the prophylactic and therapeutic experiments of antibody cocktail treatment in hamsters 4 days after SARS-CoV-2 infection

Organ	Histopathological findings	Prophylactic[1]			
		Group			
		Isotype control	Antibody cocktail 0.4 mg/kg	Antibody cocktail 4 mg/kg	Antibody cocktail 40 mg/kg
Lung					
Right (3 lobes)					
	Aggregation, alveolar macrophage, multifocal, minimal to slight[3]	4/4[4]	4/4	0/4	0/4
	Inflammation/necrosis, multifocal, minimal to moderate/severe	4/4	4/4	3/4	0/4
	Hemorrhage, multifocal, minimal to moderate	4/4	4/4	0/4	0/4
Trachea					
	Inflammation, submucosa, multifocal, minimal to slight	2/3	4/4	1/4	0/4

Organ	Histopathological findings	Therapeutic[2]			
		Group			
		Isotype control	Antibody cocktail 0.4 mg/kg	Antibody cocktail 4 mg/kg	Antibody cocktail 40 mg/kg
Lung					
Right (3 lobes)					
	Aggregation, alveolar macrophage, multifocal, minimal to slight[3]	4/4	4/4	0/4	0/4
	Inflammation or necrosis, multifocal, minimal to moderate/severe	4/4	4/4	4/4	0/4
	Hemorrhage, multifocal, minimal to slight	4/4	4/4	0/4	0/4
Trachea					
	Inflammation, submucosa, multifocal, minimal to moderate	4/4	4/4	3/4	0/4

[1]Prophylactic experiment: isotype control or antibody cocktail via intraperitoneal injection 1-day before SARS-CoV-2 infection.

[2]Therapeutic experiment: isotype control or antibody cocktail via intraperitoneal injection 3 h after SARS-CoV-2 infection.

[3] Degree of lesions was graded from one to five depending on severity: 1 = minimal (< 1%); 2 = slight (1-25%); 3 = moderate (26-50%); 4 = moderate/severe (51-75%); 5 = severe/high (76-100%).

[4] Incidence: Affected hamsters/ Total examined hamsters (n = 3-4).

Table S2. Summary of inflammatory scores of the lungs and trachea in the prophylactic and therapeutic experiments of antibody cocktail treatment in hamsters 4 days after SARS-CoV-2 infection.

Organ	Inflammatory scores	Prophylactic[1]			
		Group			
		Isotype control	Antibody cocktail 0.4 mg/kg	Antibody cocktail 4 mg/kg	Antibody cocktail 40 mg/kg
Lung					
Right (3 lobes)					
	Aggregation, alveolar macrophage, multifocal[3]	1.1±0.6[4]	1.3±0.7	0.0±0.0	0.0±0.0
	Inflammation or necrosis, multifocal	2.8±1.1	2.4±1.0	0.3±0.5[*,a]	0.0±0.0[*,a,b]
	Hemorrhage, multifocal	1.8±0.9	1.7±0.8	0.0±0.0	0.0±0.0
	Subtotal mean score[3]	1.9±1.1	1.8±1.0	0.1±0.3[*,a]	0.0±0.0[*,a,b]
Trachea					
	Inflammation, submucosa, multifocal	1.0±0.0	1.5±0.5	0.3±0.4[*,a]	0.0±0.0[*,a]

Organ	Inflammatory scores	Therapeutic[2]			
		Group			
		Isotype control	Antibody cocktail 0.4 mg/kg	Antibody cocktail 4 mg/kg	Antibody cocktail 40 mg/kg
Lung					
Right (3 lobes)					
	Aggregation, alveolar macrophage, multifocal	1.4±0.5	1.3±0.4	0.0±0.0[*,a]	0.0±0.0
	Inflammation/necrosis, multifocal	2.3±0.6	2.0±0.4	0.8±0.7[*,a]	0.0±0.0[*,a,b]
	Hemorrhage, multifocal	1.8±0.4	1.7±0.4	0.0±0.0[*,a]	0.0±0.0
	Subtotal mean score	1.8±0.6	1.7±0.5	0.3±0.6[*,a]	0.0±0.0[*,a,b]
Trachea					
	Inflammation, submucosa, multifocal	2.0±0.0	2.0±0.7	0.8±0.4[*,a]	0.0±0.0[*,a,b]

[1]Prophylactic experiment: isotype control or antibody cocktail via intraperitoneal injection 1-day before SARS-CoV-2 infection.

[2]Therapeutic experiment: isotype control or antibody cocktail via intraperitoneal injection 3 h after SARS-CoV-2 infection.

[3]The final numerical score was calculated by dividing the sum of the number per grade of affected hamsters by the total number of examined hamsters (n = 4).

[4]The subtotal mean score was calculated by dividing the sum of the number per grade of each lesion of affected hamsters by the total number of examined hamsters (n = 4).

* Statistically significant difference compared to the isotype control group each at $p<0.05$.

[a] Statistically significant difference between the antibody cocktail 0.4 mg/kg and treated groups in the prophylactic and therapeutic experiments each at $p<0.05$.

[b] Statistically significant difference between the antibody cocktail 4 mg/kg and treated groups in the prophylactic and therapeutic experiments each at $p<0.05$.

Table S3. Data collection and refinement statistics of Fab complexes

**Structure**	RBD/FI-3A	RBD/FI-3A/FD-11A	RBD/FD-5D
**PDB ID**	xxx	xxx	xxx
**Data collection**			
Space group	C2	$P3_121$	C2
Cell dimensions			
a, *b*, *c* (Å)	172.7, 147.2, 100.8	115.8, 115.8, 237.8	231.3, 146.1, 78.6
α, β, γ (°)	90, 123.8, 90	90, 90, 120	90, 103.7, 90
Resolution (Å)	84–3.00 (3.05–3.00)[a]	100–3.20 (3.30–3.26)	72–2.92 (2.97–2.92)
R_{merge}	0.181 (---)	0.263 (---)	0.284 (---)
R_{pim}	0.041 (1.554)	0.027 (0.714)	0.072 (2.231)
$I/\sigma(I)$	7.2 (0.2)	13.6 (0.3)	5.7 (0.2)
$CC_{1/2}$	0.999 (0.688)	1.000 (0.381)	0.988 (0.341)
Completeness (%)	99.4 (87.6)	100 (99.7)	99.8 (95.6)
Redundancy	20.4 (18.3)	98.9 (103.3)	16.6 (15.9)
**Refinement**			
Resolution (Å)	84–3.0	77–3.20	73–2.92
No. reflections	35090/2034	29439/1683	48402/2524
R_{work} / R_{free}	0.232/0.254	0.193/0.239	0.215/0.251
No. atoms			
Protein	9640	8065	14517
Ligand/ion/water	76	28	84
B factors (Å^2)			
Protein	178	158	127
Ligand/ion/water	110	219	167
r.m.s. deviations			
Bond lengths (Å)	0.003	0.002	0.002
Bond angles (°)	0.6	0.5	0.6

[a] Values in parentheses are for highest-resolution shell.

Table S4. CryoEM data collection statistics for FI3A.

Voltage (kV)	300
Frames	40
Dose rate (e^{-}/ Å^{2}/ s)	14.0
Total dose (e^{-}/ Å^{2})	47.7
Pixel size (Å)	0.82
Defocus (μm) [increment]	-0.8 to -2.6 [-0.3]
Symmetry	C1
Movies	4898
Particles in final reconstruction	9095 [21600]
Refined Map resolution [locally refined map] FSC = 0.143 (Å)	4.8 [6.2]
Map sharpening B-factor (Å^{2})	11.3 [-354.9]
Initial model PDB	7ND5
Model-to-map fit, CC_mask	0.6 (0.7 for S alone)
R.m.s.d., bonds (Å)	0.004
R.m.s.d., angles (°)	0.532
All-atom Clash score	4.42
Rotamer outliers (%)	4.42
Favoured (%)	96.1
Allowed (%)	3.9
Outliers (%)	0.1

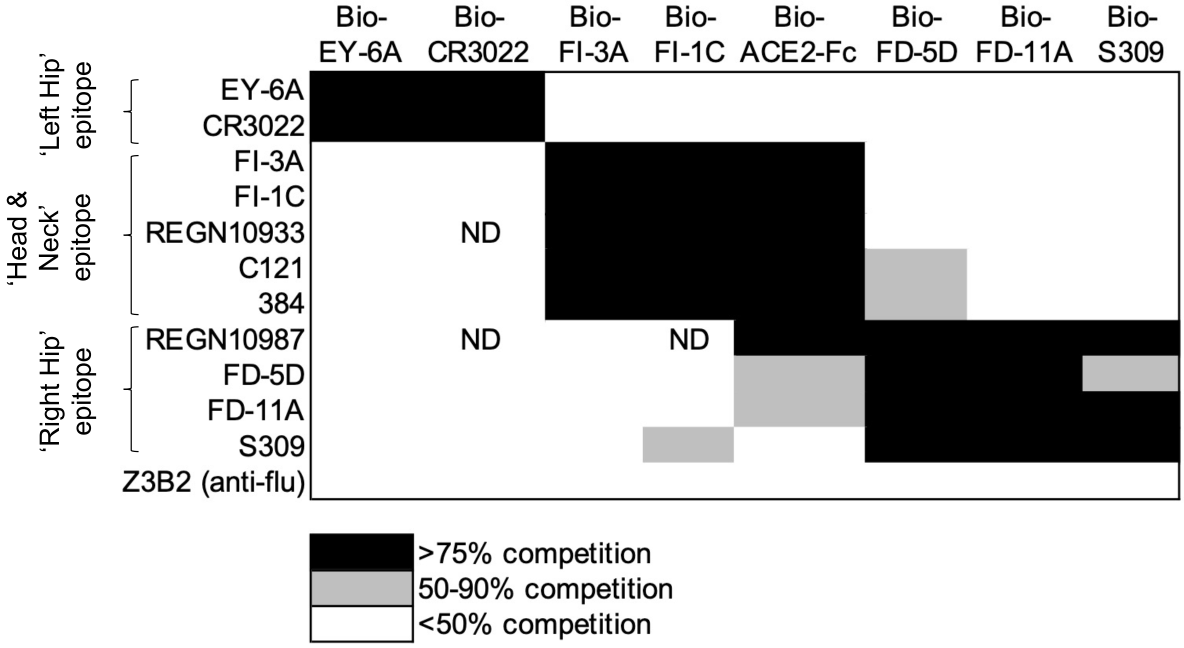

Figure S1. Non-overlapping epitopes recognized by neutralizing anti-RBD monoclonal antibodies. The grouping of neutralizing anti-RBD antibodies was based on the cross-competition analysis, the inhibition of RBD-ACE2 interaction, and structural data. Anti-RBD neutralizing monoclonal antibodies CR3022, REGN10933, REGN10987, C121, 384 and S309 were included in the experiment. Z3B2 is an anti-influenza human monoclonal antibody.

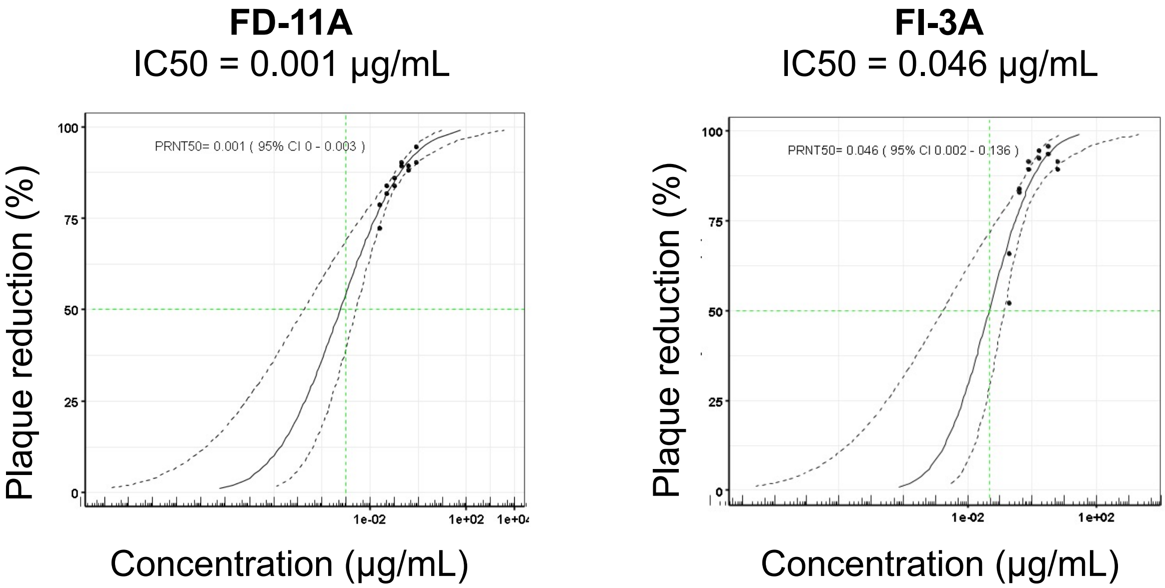

Figure S2. Neutralizing activities of FD-11A and FI-3A against wild type SARS-CoV-2, measured by plaque reduction neutralization test. The plaque reduction neutralization assay was used to examine the activity of antibody against virus. The 50% inhibitory concentrations (IC50) of FD-11A and FI-3A against SARS-CoV-2 (Australia/VIC01/2020, GenBank MT007544) were 0.001 and 0.046 µg/mL, respectively. Three technical replicates were done and the confidence limits were calculated.

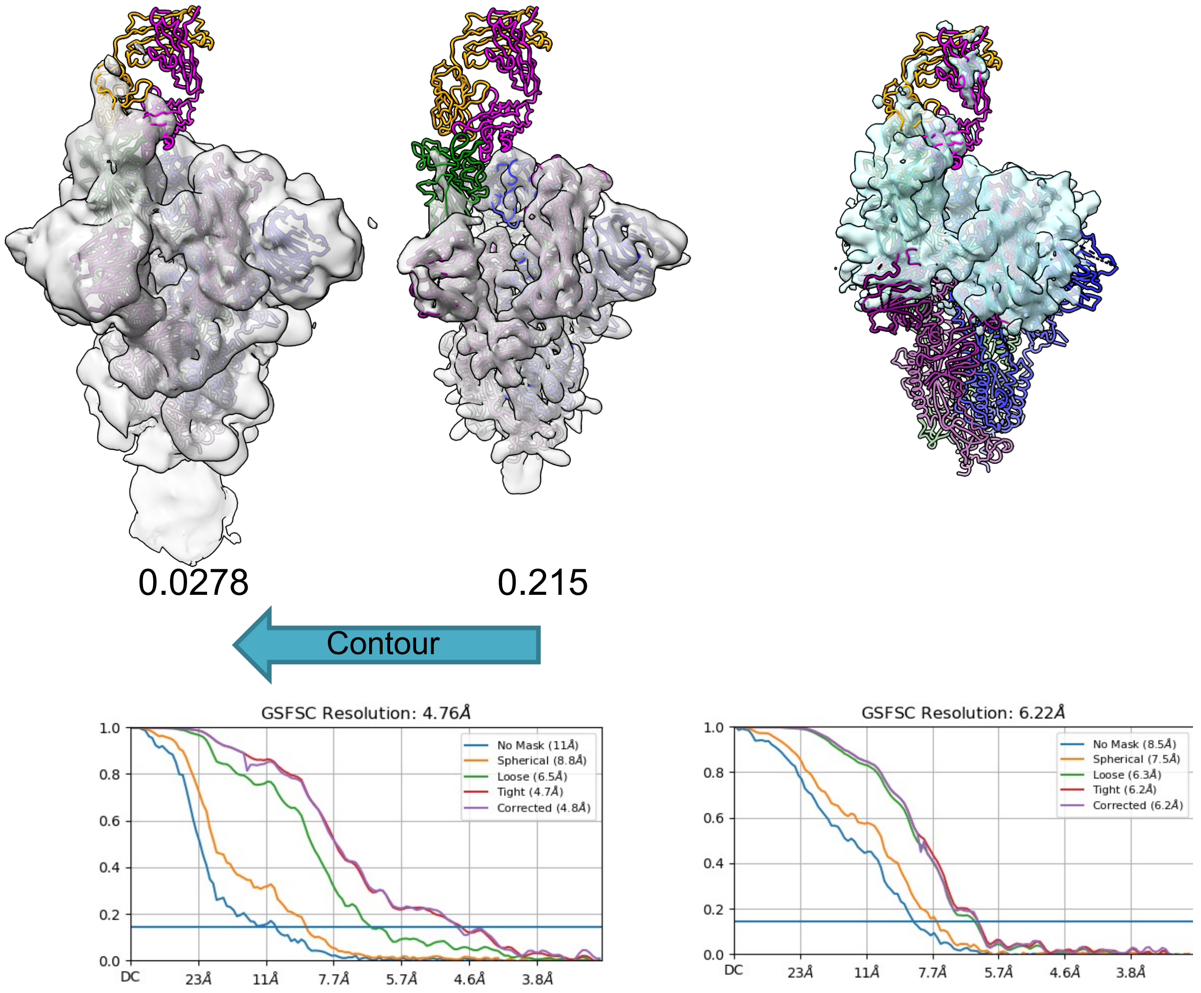

Figure S3. Low resolution cryo-EM reconstructed volumes of FI-3A in complex with Spike. Left: entire spike reconstruction (grey) at low and high contour levels, respectively with a model of spike with RBD up and FI-3A colored by chain; Right: Map from local refinement of the RBD plus fab region. Associated FSC curves are shown below each structure. The model shown with each volume is of FI-3A with RBD was superimposed with the up RBD from a rigid body fitted model, PDB 6z97 colored by chain. FI-3A fab is shown in orange and magenta.

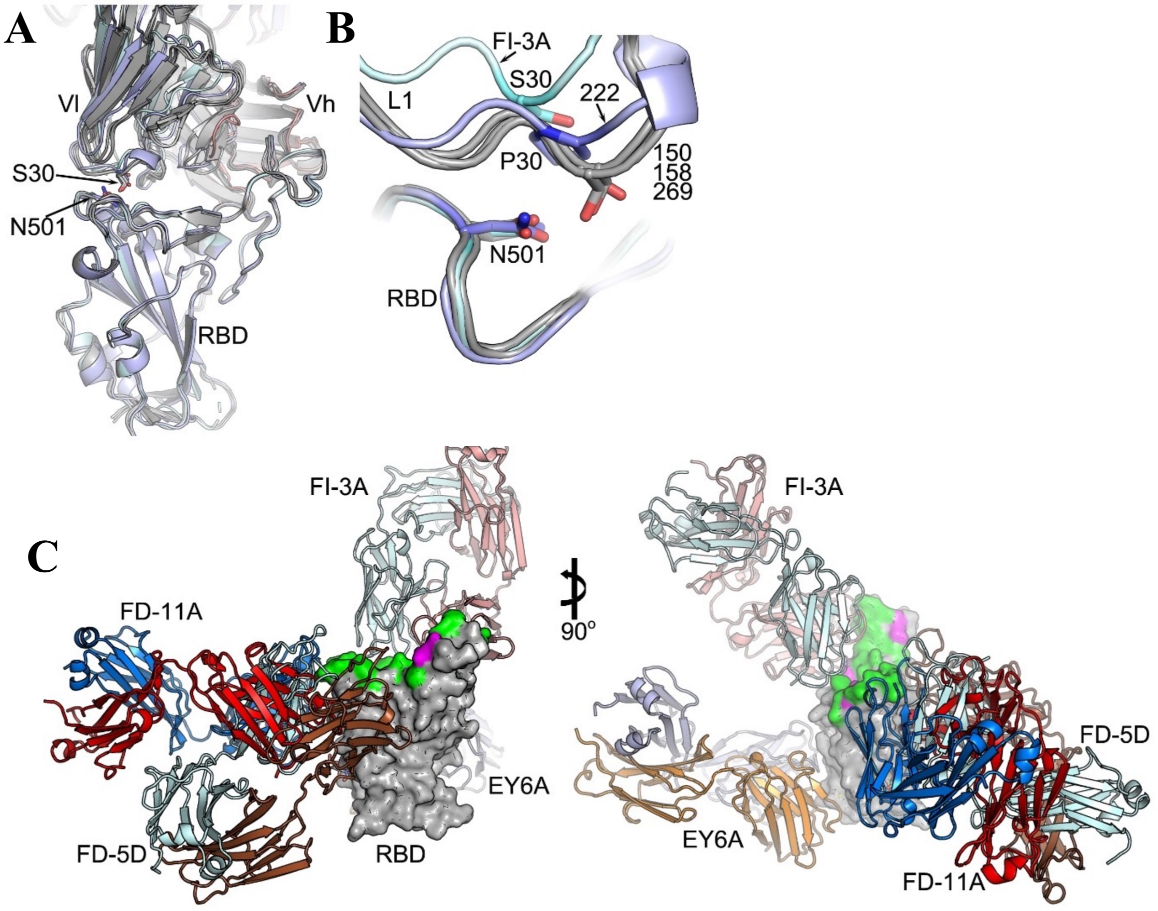

Figure S4. Relative binding position and orientation of the four RBD binding Fabs. A, The binding mode of FI-3A compared with that of COVOX-222, 150, 158 and 269. The RBD and the LC are in cyan, and HC is in salmon for RBD/FI-3A complex, RBD/fab-222 is in light blue, and others in grey. **B**, Closeup of (**A**) at N501 of the RBD and CDR-L1 interface. **C**, Orthogonal view of the binding modes of the four RBD binding Fabs from our set of mAbs whose complex structure with RBD were determined. The RBDs in the complexes were overlapped and one is shown as grey surface with ACE2 footprint marked in green, and the mutation sites (K417, E484 and N501) of P.1 and B.1.351 variants in magenta. Vl, variable domain of light chain; Vh, variable domain of heavy chain.

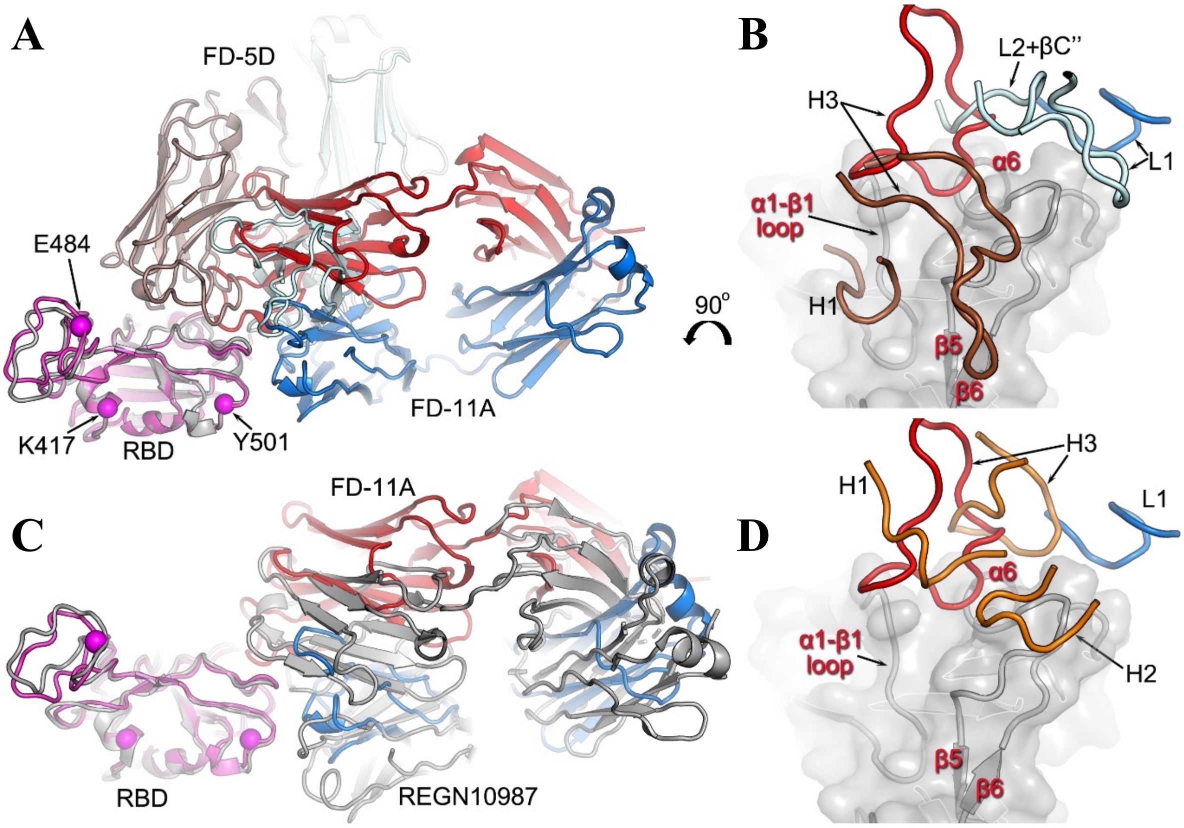

Figure S5. Comparison of the binding modes of FD-5D and FD-11A. A, Comparing the binding modes of FD-11A and FD-5D by overlapping the RBD. The RBD, the HC and LC of the RBD/FD-11A complex are colored in magenta, red and blue respectively, while the corresponding chains of the RBD/FD-5D complex are drawn in grey, brown and pale cyan. The mutation sites of the P.1 and B.1.351 variants are marked with magenta spheres. **B**, The CDR loops of FD-11A and FD-5D that involved in interactions with the RBD and colored as in (**A**). **C**, Comparison of the binding mode of FD-11A (same colors as in (**A**)) with that of REGN10987 (grey). **D**, The CDR loops in (**C**) involved in interactions with the RBD. Only the HC of REGN10987 has direct contacts with the RBD and the CDR loops in (**D**) are colored in orange. HC, heavy chain; LC, light chain.

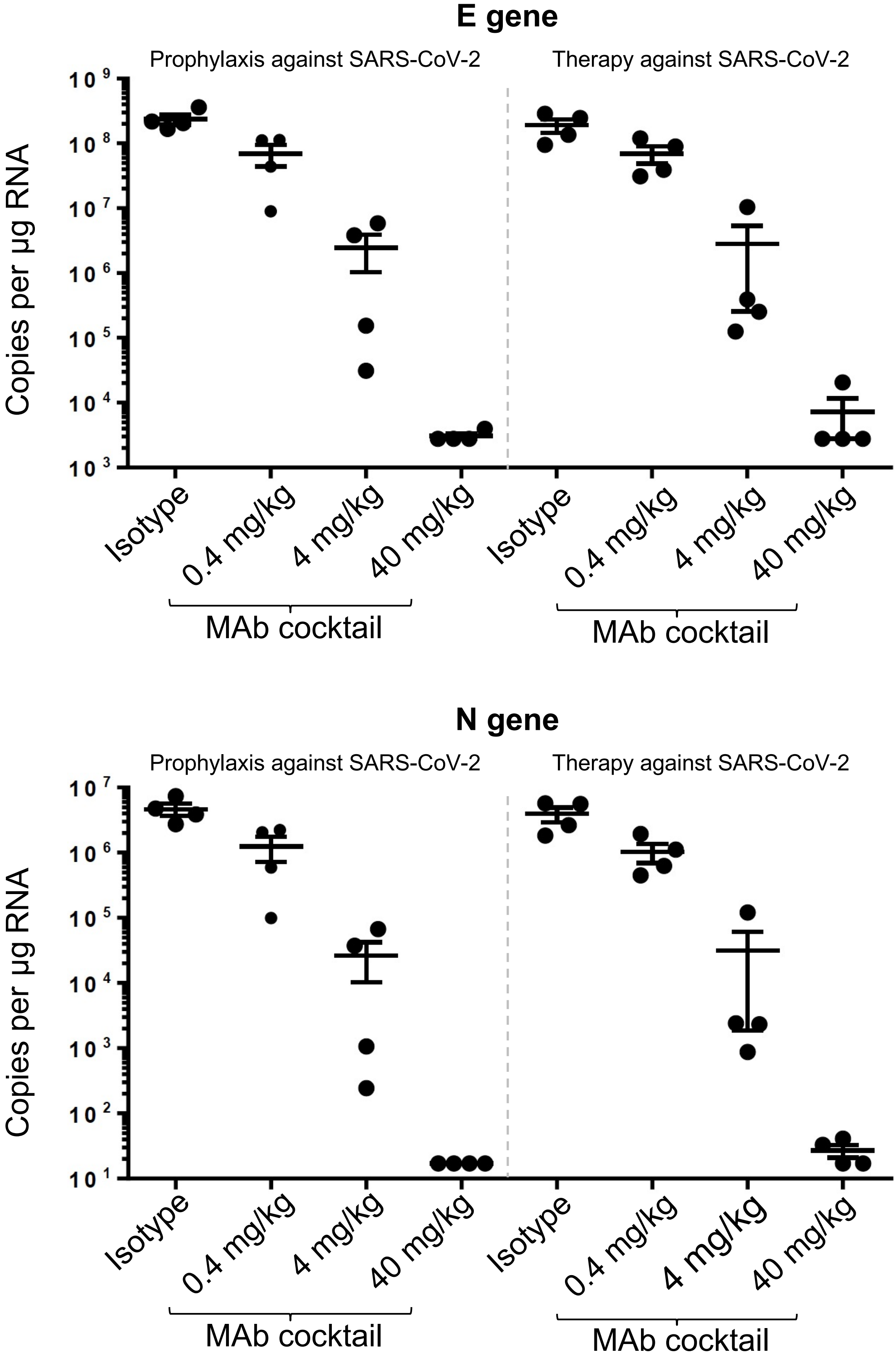

Figure S6. Viral RNA (copies per µg RNA) detected in the lungs of hamsters challenged with SARS-CoV-2 (n = 4 per group) at day 4 post challenge. Viral loads were determined by quantitative reverse transcription PCR for detection of SARS-CoV-2 E and N genes. The error bars represent standard deviations of the mean. E, envelop; N, nucleocapsid.

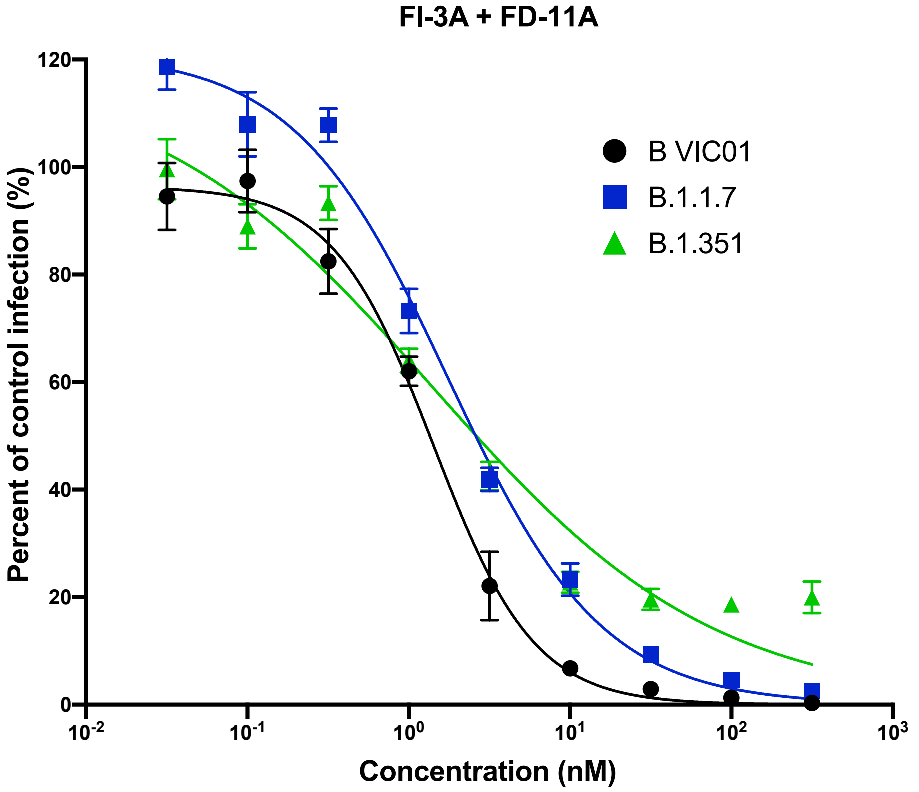

Figure S7. Neutralization of antibody cocktail FD-11A plus FI-3A cocktail against SARS-CoV-2 UK B.1.1.7 and South African B.1.351 variants. The microneutralizaton assay was used to determine the activity of antibody against wild type SARS-CoV-2. Data were normalized to control (no antibody) values of foci and individual points are displayed ± 1 standard deviation of mean, and curves are shown for antibody cocktail fitted the standard dose-response (Hill) equation (n=4). The 50% inhibitory concentrations of FD-11A plus FI-3A cocktail against SARS-CoV-2 (Australia/VIC01/2020, B VIC01 GenBank MT007544), B.1.1.7 (20I/501Y.V1.HMPP1, H204820430, 2/UK/VUI/1/2020) and B.1.351 (20I/501.V2.HV001) were 0.214, 0.264 and 0.211 μg/mL, respectively.

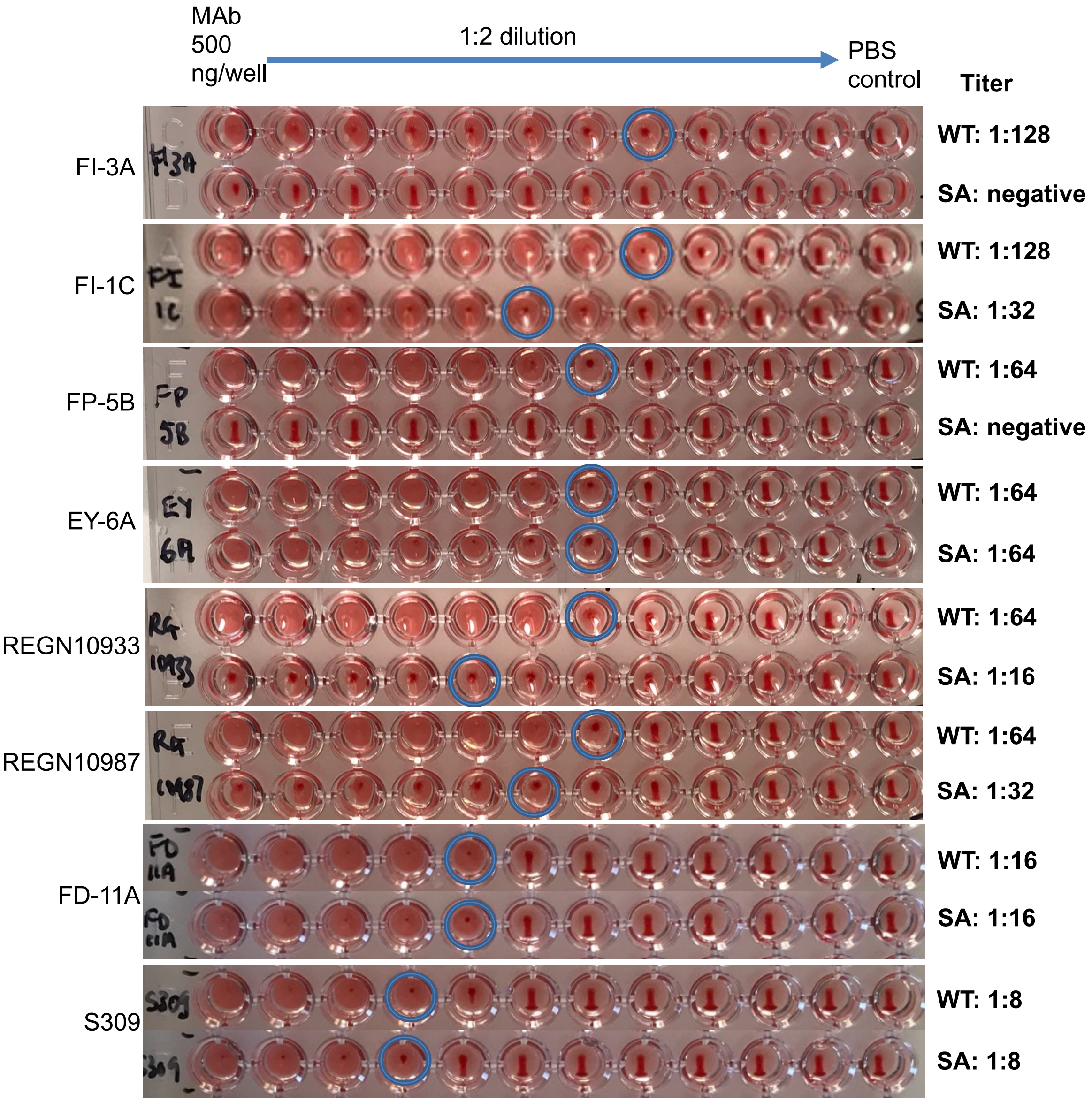

Figure S8. The activity of anti-RBD antibodies with IH4-RBD of wild type SARS-CoV-2 and South African B.1.351 variants in the hemagglutination assay. In the assay, RBD-bound antibodies are able to cross-link IH4-RBD and lead to the agglutination. Positive agglutination endpoints (loss of teardrop) are marked with a blue solid-line circle. FI-3A and FP-5B lost reactivity with RBD of South African B.1.351 variant. WT, wild type; SA, South African B.1.351 lineage.

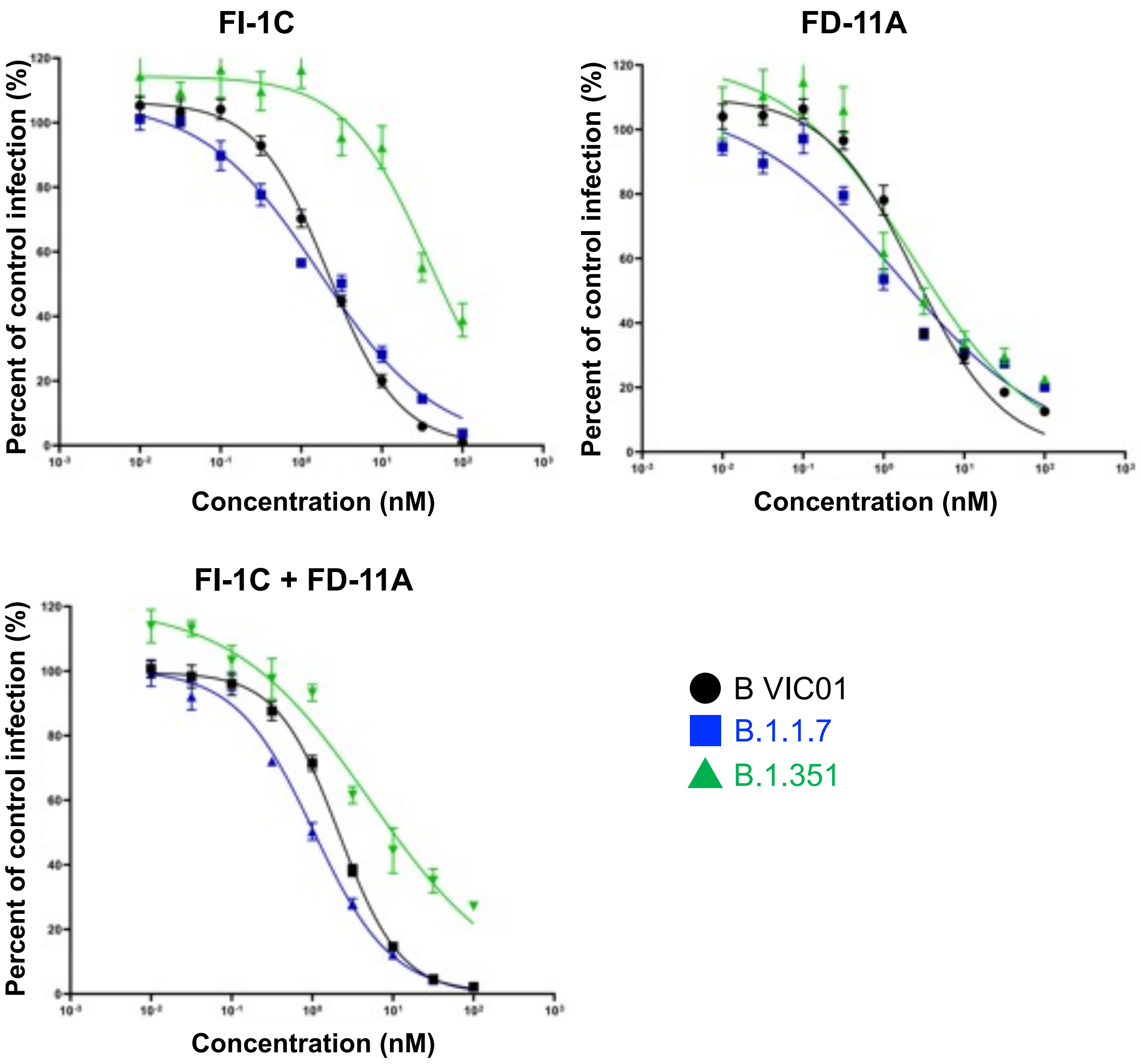

Figure S9. Neutralization of antibody cocktail FI-1C plus FD-11A cocktail against SARS-CoV-2 South African B.1.351 variant. The microneutralization assay was used to determine the activity of antibody against wild type SARS-CoV-2. Data were normalized to control (no antibody) values of foci and individual points are displayed ± 1 standard deviation of mean, and curves are shown for antibody cocktail fitted the standard dose-response (Hill) equation (n=4). The 50% inhibitory concentrations of FI-1C against SARS-CoV-2 (Australia/VIC01/2020, B VIC01 GenBank MT007544), B.1.1.7 (20I/501Y.V1.HMPP1, H204820430, 2/UK/VUI/1/2020) and B.1.351 (20I/501.V2.HV001) were 0.324, 0.254 and 5.7 µg/mL, respectively. The 50% inhibitory concentrations of FD-11A plus FI-1C cocktail against SARS-CoV-2 VIC01, B.1.1.7 and B.1.351 were 0.320, 0.156 and 0.769 µg/mL, respectively.

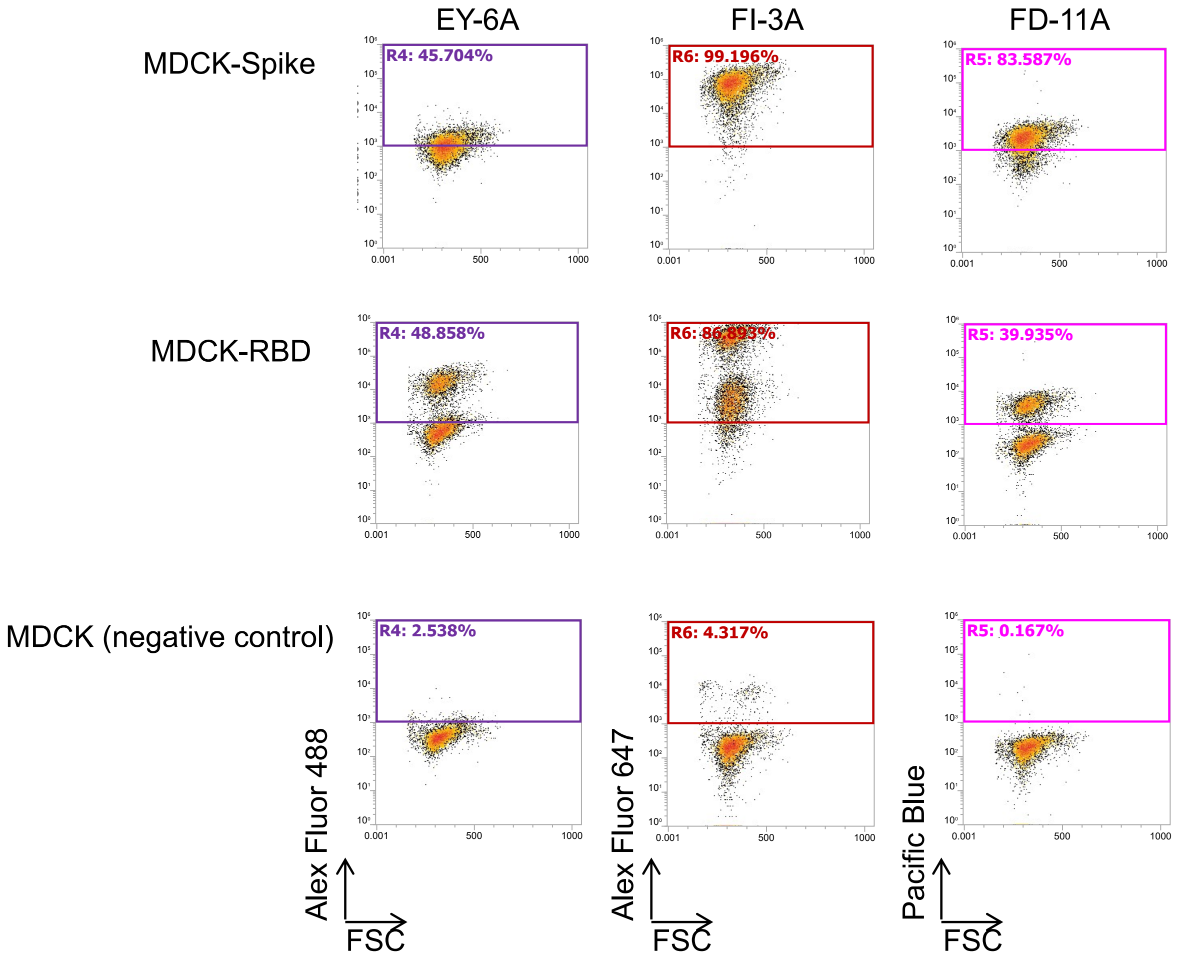

Figure S10. Simultaneous binding of antibodies EY-6A, FD-11A, and FI-3A cocktail on to the SARS-CoV-2 RBD and spike proteins expressed on the MDCK cell in the flow cytometry.
